# Supplementary material for: Ongoing Network State Controls the Length of Sleep Spindles via Inhibitory Activity
Source: Neuron. 2014 Jun 18;82(6):1367–79. doi: 10.1016/j.neuron.2014.04.046 (PMC4064116; doi:10.1016/j.neuron.2014.04.046)
Supplement: Document S1. Figures S1–S8 [file mmc1.pdf]

Neuron, Volume *82*

Supplemental Information

Ongoing Network State Controls the Length of Sleep Spindles via Inhibitory Activity

Péter Barthó, Andrea Slézia, Ferenc Mátyás, Lejla Faradzs-Zade, István Ulbert, Kenneth D. Harris, and László Acsády

Supplementary Figures

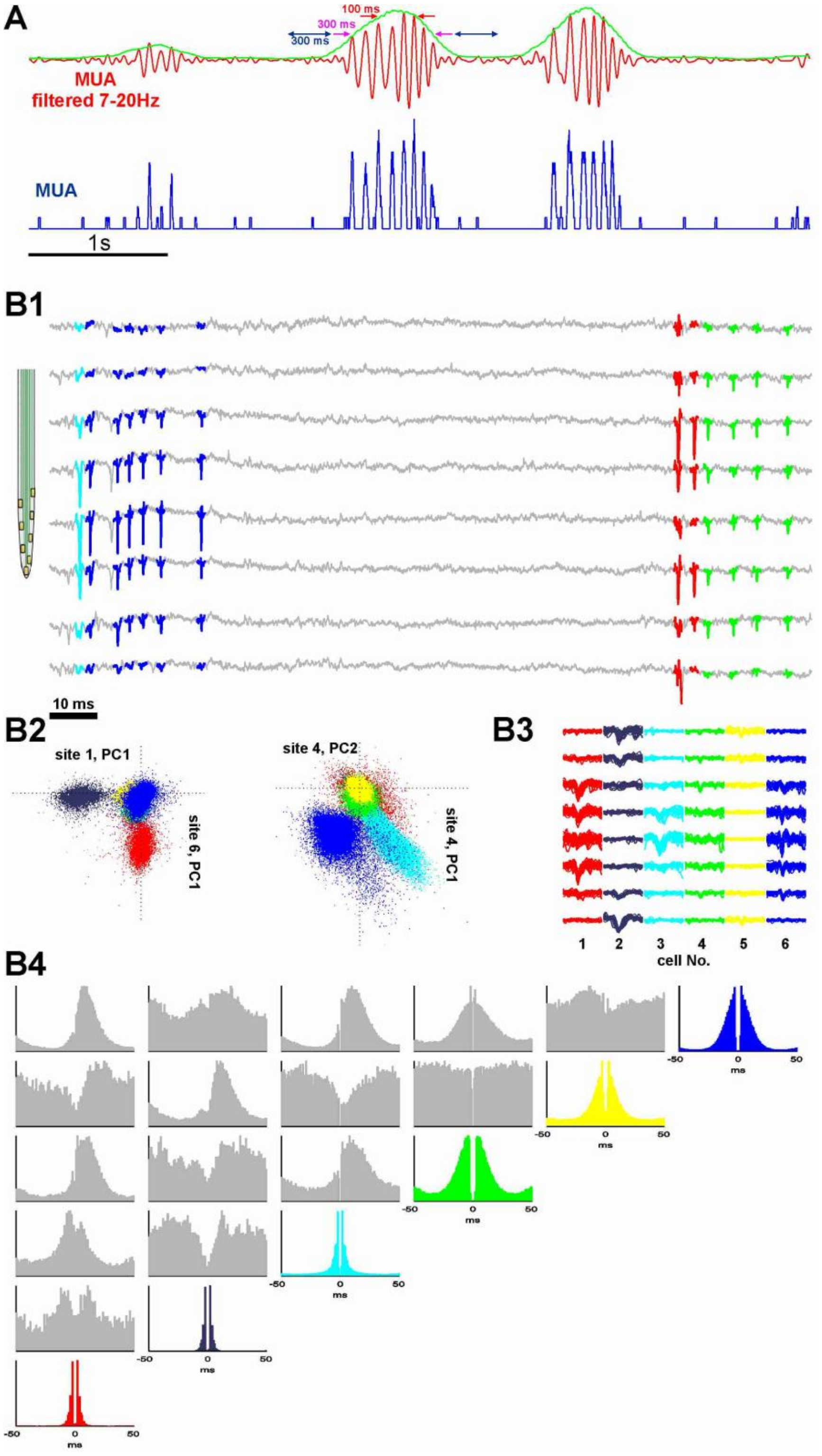

Supplementary Figure 1, related to Figure 1. Spindle detection and unit clustering

A) Detection of multiunit spindles in the thalamus. Blue trace, local thalamic multiunit activity smoothed with a 11 ms long square kernel; red trace, the same trace filtered between 7-20 Hz; green trace, envelope fitted on red trace, used for spindle detection. Criteria for spindles were: the envelope should exceed 1.5 standard deviations of fMUA for >300ms (magenta arrow), with a central portion of the envelope exceeding 2.5*SD for >100 ms (red arrow), and a relative silence below 1.5 STD for 300 ms before and after the spindle (blue arrow). **B**) Overview of the spike clustering procedure**.** (**B1**) Raw trace from VB recorded by the eight recording sites of on one shank (left) of the four-shank octrode. Spike waveforms of four different neurons can be distinguished during the period shown. (**B2**) Two-dimensional projections of the clustered action potentials from the same shank. (**B3**) Spike waveforms of the clustered neurons. (**B4**) Auto- and crosscorrelograms of the same cells. Note the different shapes of the autocorrelogram in case of units 1-2-3 vs. 4-5-6, and the asymmetric cross-correlation between the two groups.

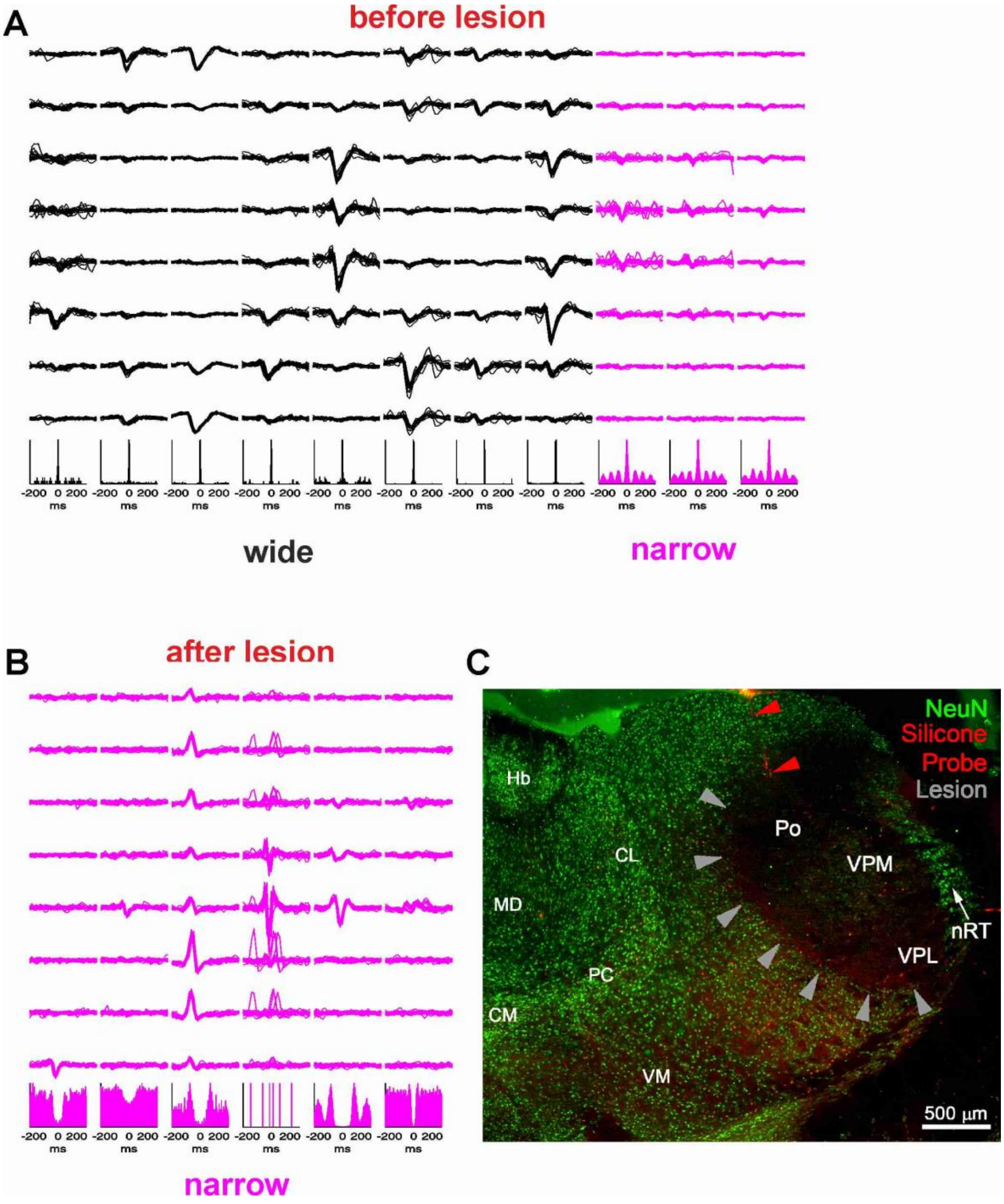

Supplementary Figure 2, related to Figure 3. Wide spikes selectively disappear from VB after focal kainic acid lesion.

(**A**) Spike waveforms on the eight recording sites of wide spikes (black, 8 units) and narrow spikes (magenta, 3 units) recorded by one of the shanks inserted into the VB before the lesion. The autocorrelograms of each unit are shown at the bottom of the columns. Note pronounced modulation of narrow spikes at the spindle frequency range. (**B**) Four hours after the KA lesion only narrow spike units can be recorded at the same location (silicon probe not moved). Spindle modulation disappears from the autocorrelograms (**C**) NeuN staining shows complete absence of thalamocortical cells (grey arrowheads) after iontophoresis of kainic acid into VB. Red arrowheads; electrode track.

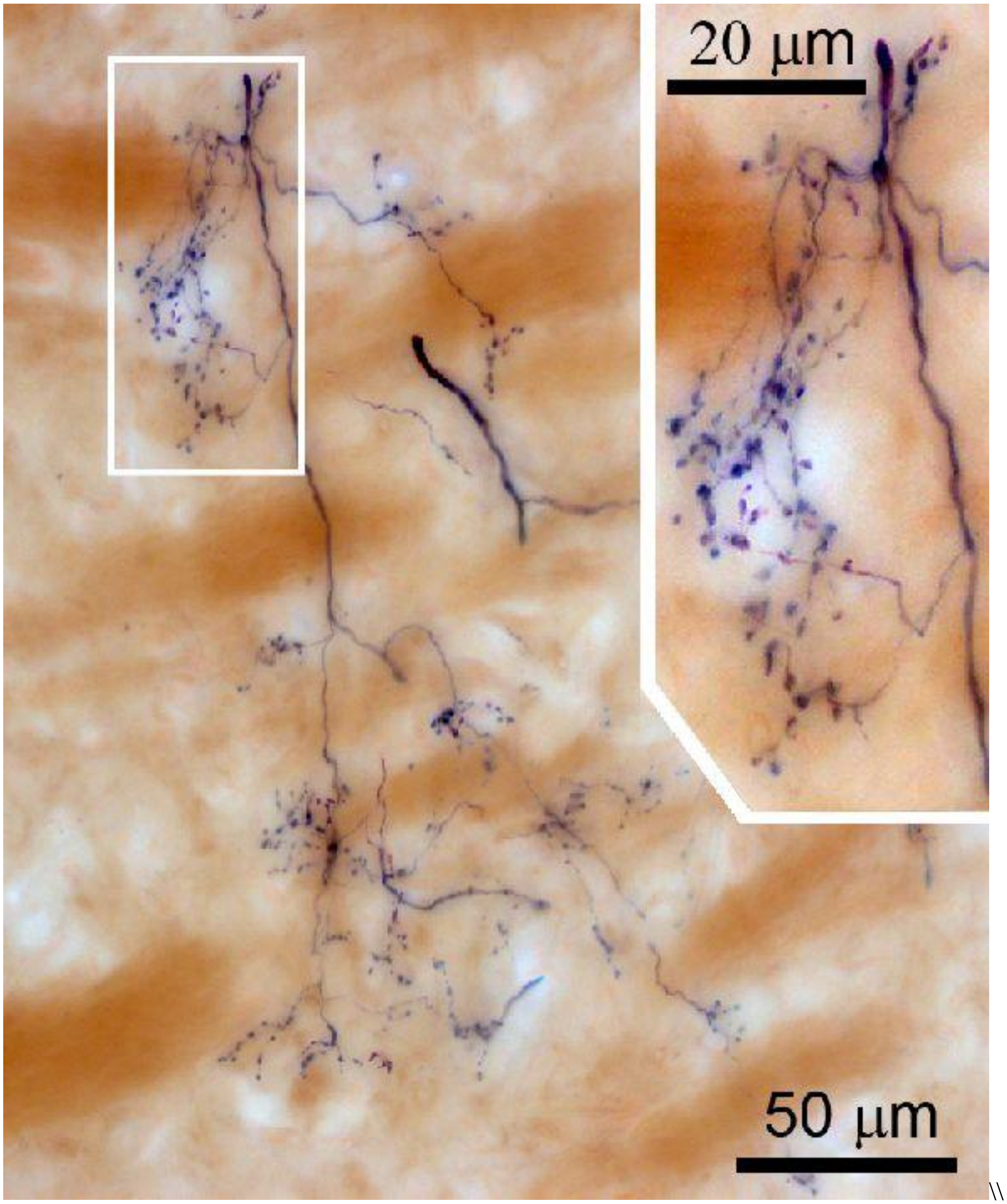

\\

Supplementary Figure 3, related to Figure 3. Dense axon terminal clusters of nRT cells in VB.
Part of the axon terminal arbor of a juxtacellularly filled nRT neuron in VB. Note the extremely dense clusters of axon terminals. The right inset shows that within a reasonably small volume of neuropyl (>20 micron) close to 100 terminals of the same axon branch can be present. Since this terminal cluster fire nearly simultaneously, we predict that it generates a large enough extracellular field response which can be recorded by several recording sites of the same electrode shank.

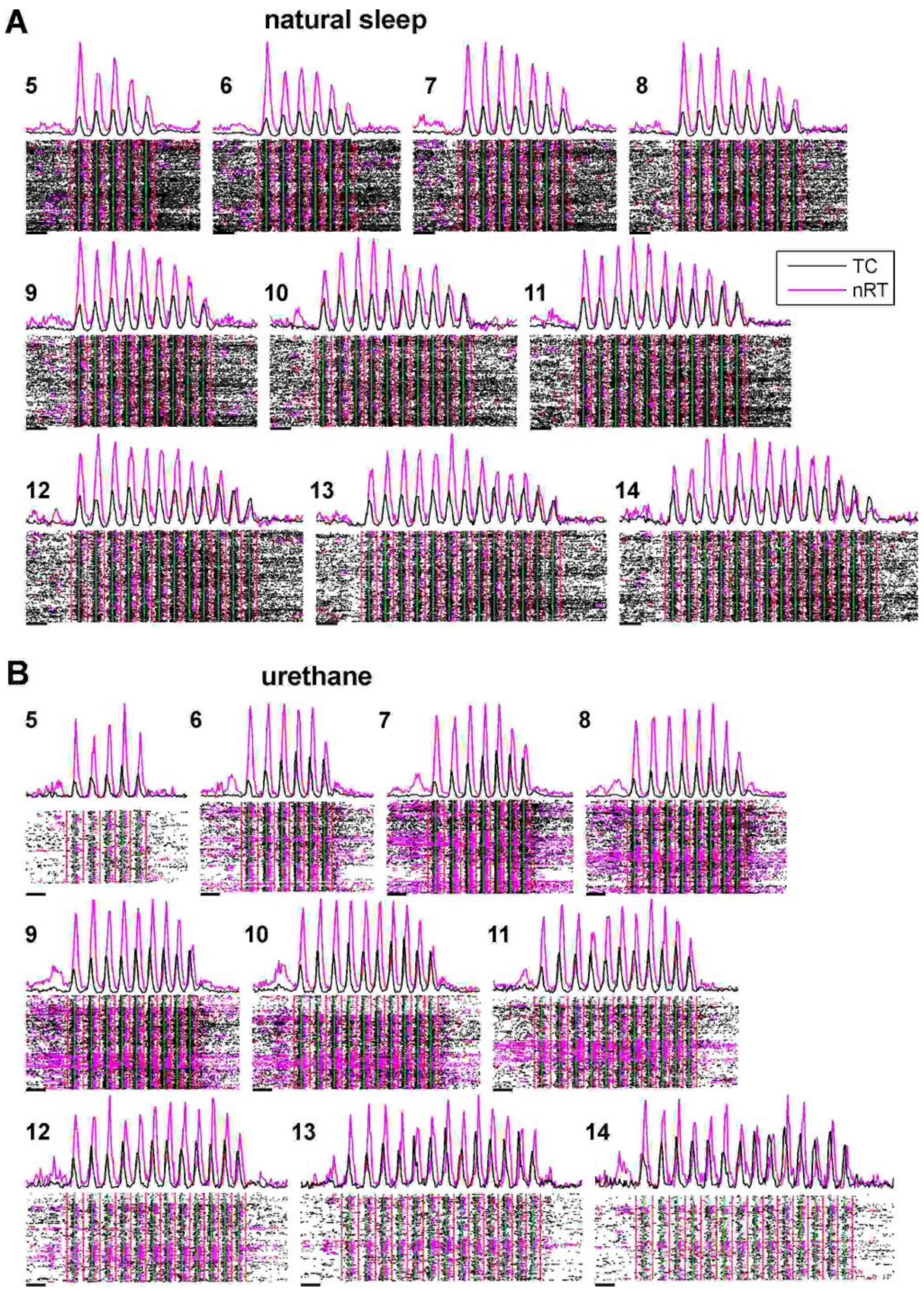

Supplementary Figure 4, related to Figure 5. Network activity of TC and nRT cells during natural sleep and urethane anesthesia.

Perievent time histograms (PSTH) and rasterplots of TC (black) and nRT (magenta) activity during spindles for all lengths under natural sleep (**A**), and urethane anesthesia (**B**). PSTHs are aligned to the peak of the cycles for visualisation purposes (here and Fig3A only). The plots are arranged from short spindles (5 cycles top left) to long spindles (14 cycles) bottom right. The plots display recordings from several animals and recording locations. Note that in both conditions short spindles start with high nRT actvity which decreases cycle to cycle. This scenario changes progressively to lower initial nRT activity with increasing spindle length. Scales: 100ms

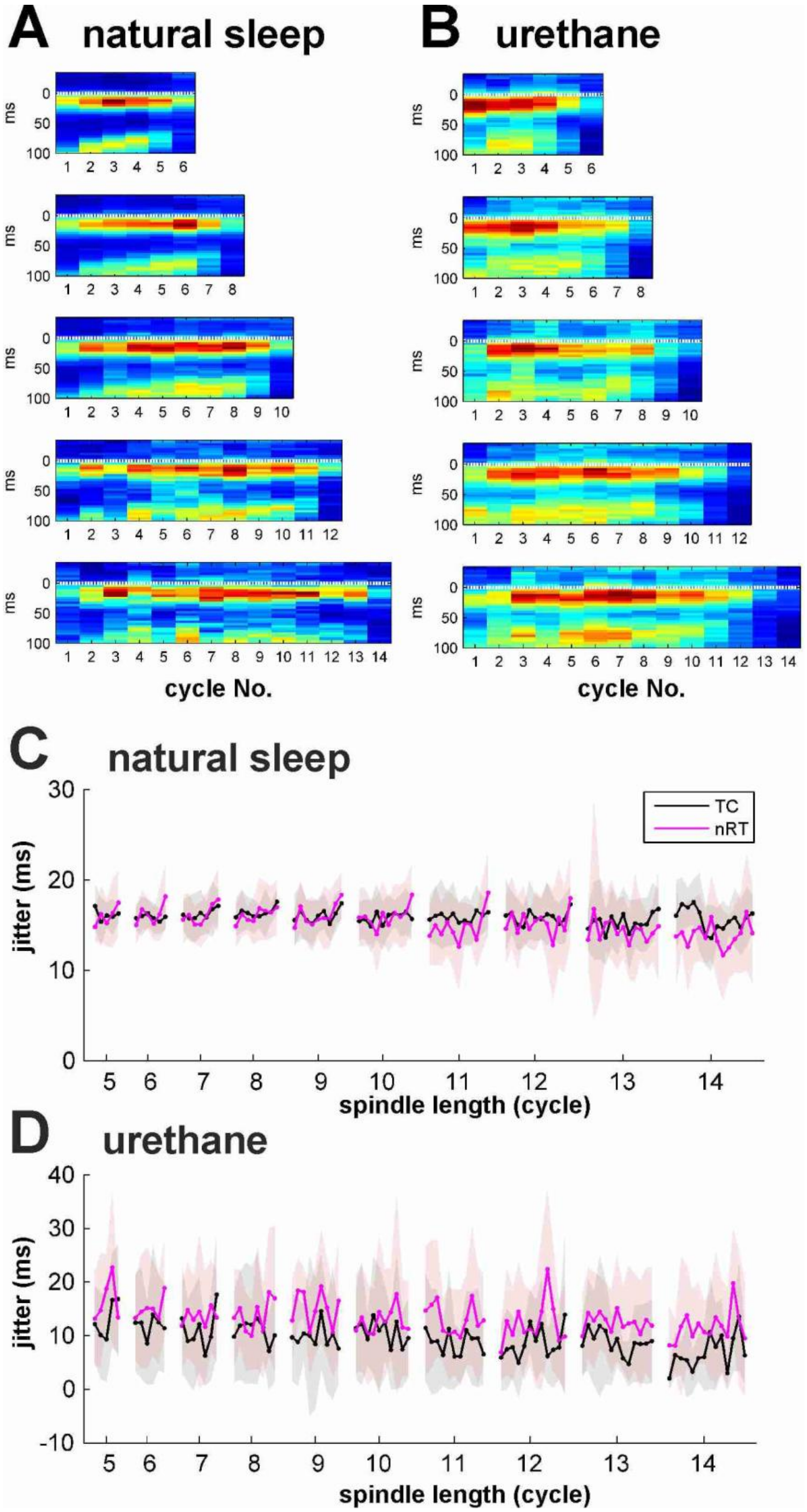

Supplementary Figure 5, related to Figure 5. Stable cross correlation and jitter of TC and nRT units.

A-B) Summed cycle-by-cycle cross-correlograms between TC and nRT cells for several (6,8,10,12,14 cycles) spindle lengths in naturally sleeping (**a**), and urethane anesthetized (**b**) animals. Warm colors indicate higher values. TC cells always precede nRT with a stable delay despite the changes in the number of spikes fired. **C-D)** Cycle-by-cycle change in jitter measured as SD of action potential distance from peak in TC and nRT cells for all spindle lengths in naturally sleeping (**A**), and urethane anesthetized (**B**) animals. Shading

indicates SEM.

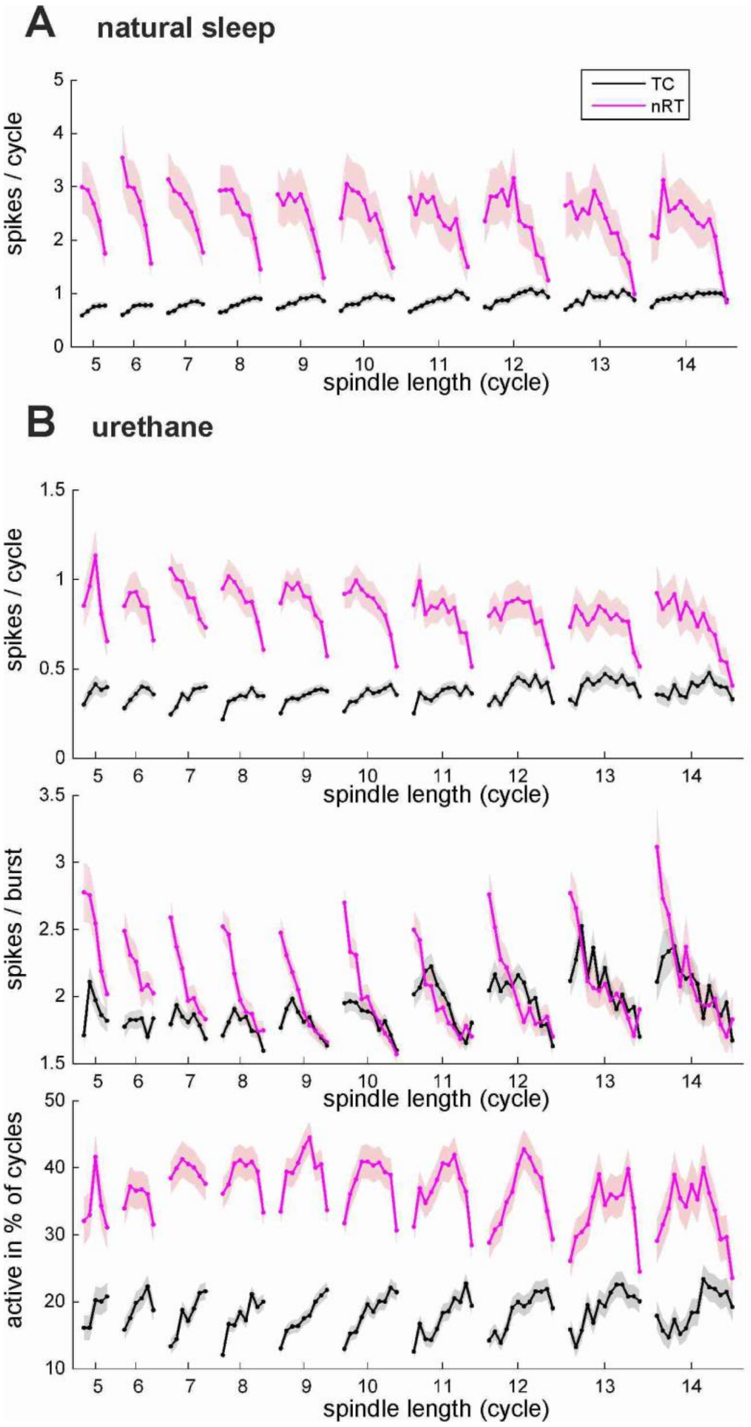

Supplementary Figure 6, related to Figures 5,6. Cycle-by-cycle neuronal activity during spindles of natural sleep and under urethane anesthesia.

(**A**) Mean number of spikes per cycle for all spindle lengths under natural sleep for TC cells (black), nRT cells (magenta) and the ratio of the two activities (red). Note the increasing ratio of excitatory / inhibitory actvity throughout the spindles (**B**) Mean number of spikes per cycle, number of spikes per burst, and firing probability for all spindle lengths under urethane anesthesia. Compare to (**A**) and Figure 4. Shading indicates SEM.

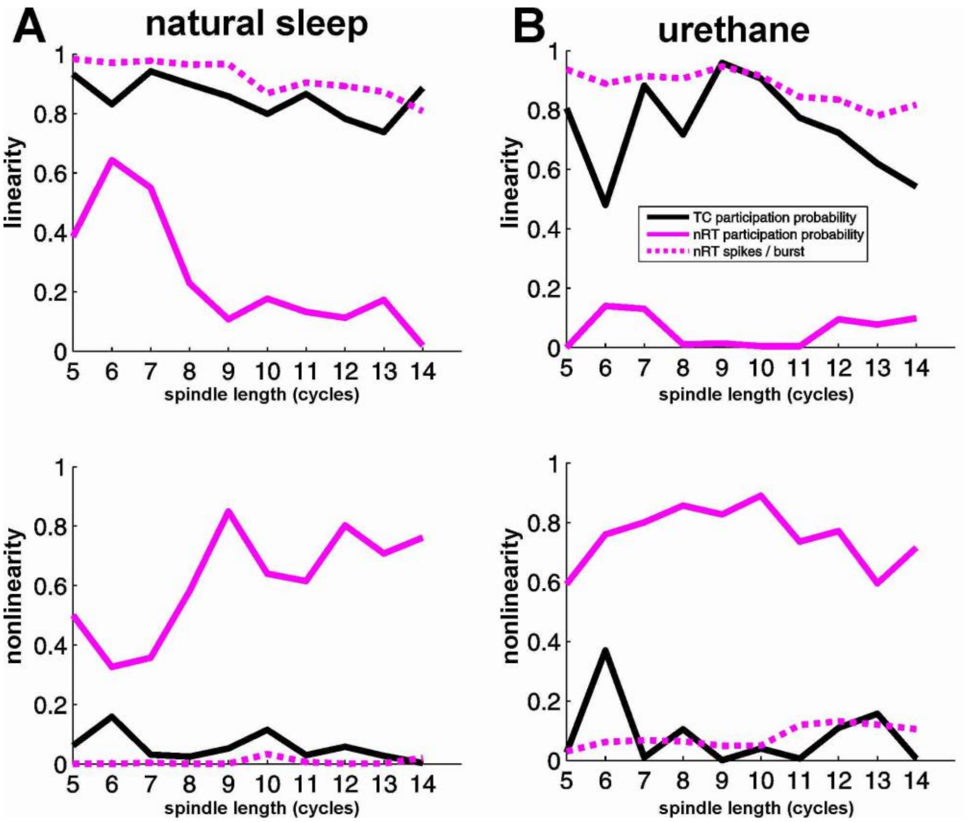

Supplementary Figure 7, related to Figure 6.. Shape analysis of trajectories

(**A**) Linearity and nonlinearity of curves for all spindle lengths on Figs. 6 and S8. Linearity was defined as R^2_{linear}, nonlinearity as $R^2_{linear} - R^2_{quadratic}$, for linear and quadratic fits of the curve, respectively. TC participation probability and nRT spikes/burst are linear in all groups, while nRT participation probability is more linear for short spindles (<8 cycles), and more nonlinear for long spindles. TC spikes/burst was omitted for having too low variance explained. (**B**) Same curves under urethane anesthesia. Note that nRT participation probability is nonlinear even for short spindles.

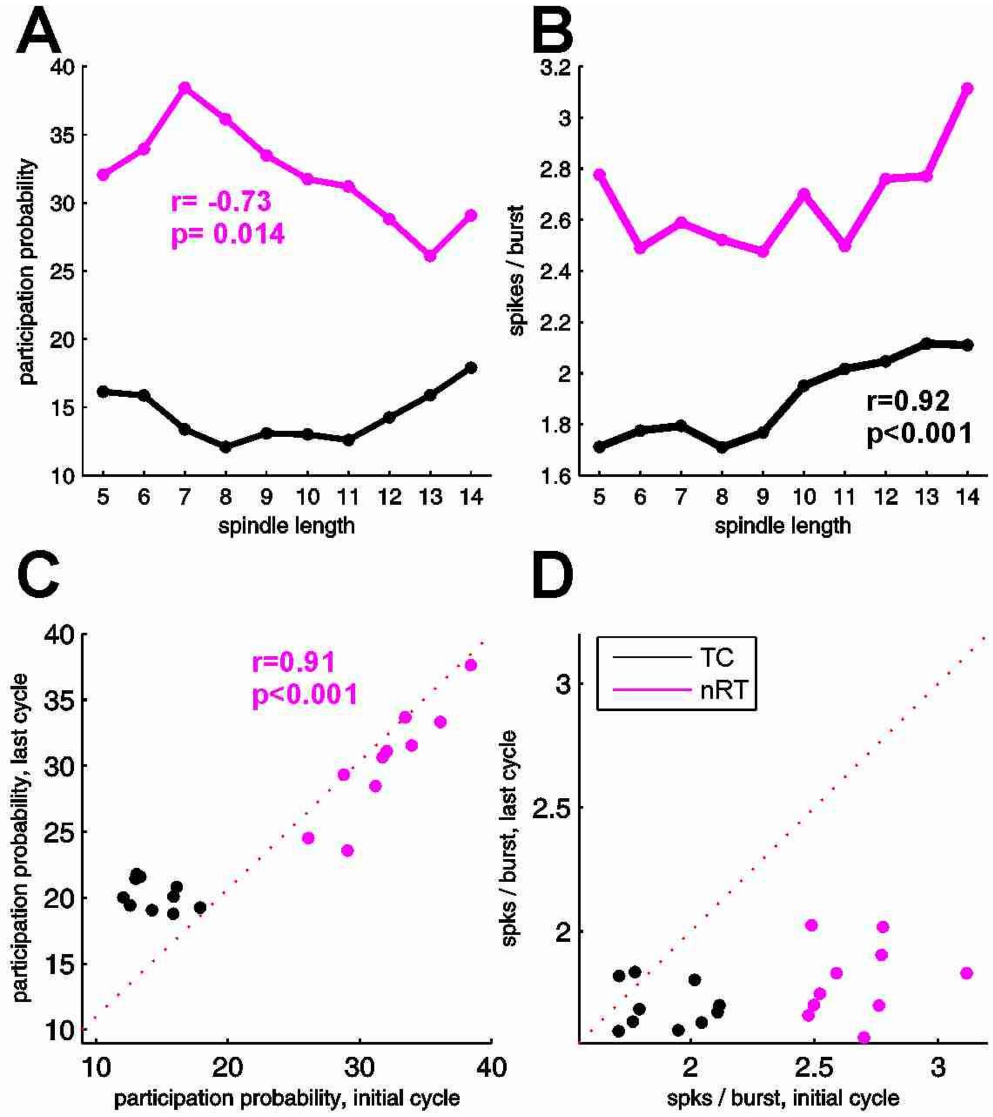

Supplementary Figure 8, related to Figure 7. Initial network state correlates with spindle length under urethane anesthesia

(**A-D**) Same as on Figure 7. Initial nRT participation probability and TC spikes/burst show significant correlation with spindle length. Initial and final nRT participation probability also correlates significantly.
