# Supplementary material for: Ongoing Network State Controls the Length of Sleep Spindles via Inhibitory Activity
Source: Neuron. 2014 Jun 18;82(6):1367–79. doi: 10.1016/j.neuron.2014.04.046 (PMC4064116; doi:10.1016/j.neuron.2014.04.046)
Supplement: Document S2. Article plus Supplemental Information [file mmc2.pdf]

Neuron
Article

Ongoing Network State Controls the Length of Sleep Spindles via Inhibitory Activity

Péter Barthó,[1,*] Andrea Slézia,[1] Ferenc Mátyás,[1] Lejla Faradzs-Zade,[1] István Ulbert,[2,3] Kenneth D. Harris,[4] and László Acsády[1,*]
[1]Laboratory of Thalamus Research, Institute of Experimental Medicine, Hungarian Academy of Sciences, 1083, Budapest, 43 Szigony utca, Hungary
[2]Institute of Cognitive Neuroscience and Psychology, Research Centre for Natural Sciences, Hungarian Academy of Sciences, 1083, Budapest, 1068, 83-85 Szondi utca, Hungary
[3]Péter Pázmány Catholic University, Faculty of Information Technology and Bionics, 1083, Budapest, 50/A Práter utca, Hungary
[4]UCL Institute of Neurology, UCL Department of Neuroscience, Physiology, and Pharmacology, 21 University Street, London WC1E 6DE, UK
*Correspondence: bartho@koki.hu (P.B.), acsady@koki.hu (L.A.)
http://dx.doi.org/10.1016/j.neuron.2014.04.046
This is an open access article under the CC BY license (http://creativecommons.org/licenses/by/3.0/).

SUMMARY

Sleep spindles are major transient oscillations of the mammalian brain. Spindles are generated in the thalamus; however, what determines their duration is presently unclear. Here, we measured somatic activity of excitatory thalamocortical (TC) cells together with axonal activity of reciprocally coupled inhibitory reticular thalamic cells (nRTs) and quantified cycle-by-cycle alterations in their firing in vivo. We found that spindles with different durations were paralleled by distinct nRT activity, and nRT firing sharply dropped before the termination of all spindles. Both initial nRT and TC activity was correlated with spindle length, but nRT correlation was more robust. Analysis of spindles evoked by optogenetic activation of nRT showed that spindle probability, but not spindle length, was determined by the strength of the light stimulus. Our data indicate that during natural sleep a dynamically fluctuating thalamocortical network controls the duration of sleep spindles via the major inhibitory element of the circuits, the nRT.

INTRODUCTION

The large-scale activity of the brain is organized by a great variety of network oscillations, which temporally bind the activity of distinct cell populations. Although a wealth of data indicates a role of inhibitory GABAergic cells in pacing the frequency of oscillations (Buzsáki, 2006), the mechanisms controlling the duration and termination of oscillatory events are still mysterious. A major brain oscillation with variable length is the sleep spindle. These 1- to 3-s-long transient events have a frequency of 7–15 Hz and are most prevalent during stage II sleep. Appropriate regulation of spindle density and duration is critical to proper brain function. Spindle density shows strong correlation with memory performance (Fogel et al., 2007), problem-solving ability, and the general intelligence of an individual (Bódizs et al., 2005). Both the incidence and duration of spindles increase following learning (Morin et al., 2008) and decrease with age (Nicolas et al., 2001). Aberrant spindle-like activity is believed to underlie absence epilepsy (Avanzini et al., 2000; Huguenard and McCormick, 2007; Kostopoulos, 2000; Picard et al., 2007). Extremely long spindles characterize mental retardation in childhood (Gibbs and Gibbs, 1962; Shibagaki et al., 1982). Schizophrenia on the other hand is associated with a marked reduction of spindle length (Ferrarelli et al., 2007).

Previous studies (von Krosigk et al., 1993; Steriade and Deschenes, 1984; Steriade et al., 1985) have suggested that spindles are generated in the thalamus, through a rhythmic interaction of excitatory thalamocortical (TC) neurons and inhibitory neurons of the nucleus reticularis thalami (nRT), that in turn entrains cortical activity. In this model, synchronized bursts of nRT neurons cause prolonged inhibition in TC cells, which deinactivate low-threshold Ca^{2+} (It) channels and induce TC cells to fire a rebound burst upon IPSP termination. This drives a new nRT burst and the next oscillation cycle begins.

Several candidate mechanisms have been proposed to control the termination of sleep spindles. These data indicated a progressive change of the intrinsic properties of either TC (Bal and McCormick, 1996; Lüthi and McCormick, 1998; Lüthi et al., 1998) or nRT cells (Bal et al., 1995a; Kim and McCormick, 1998) during the spindles leading to stop burst generation and the initiation of a next cycle. According to another proposal, spindles terminate due to disruption of the synchronization of TC-nRT network activity, caused by an increase of poorly timed cortical input as the spindle progresses (Bonjean et al., 2011; Timofeev et al., 2001). These proposals make testable predictions for how TC and nRT cells alter their firing activity during the progression of a spindle. However, testing these alternative scenarios experimentally has so far remained elusive, due to the challenge of simultaneously recording topographically coupled populations of TC and nRT cells in freely sleeping animals. As a consequence the factors controlling the duration of spindles in vivo—critically correlated with several neuropsychiatric disorders—remained unclear.

In the present study, we performed simultaneous recording of topographically coupled TC and nRT cells in freely sleeping rats and quantified their activity on a cycle-to-cycle basis during

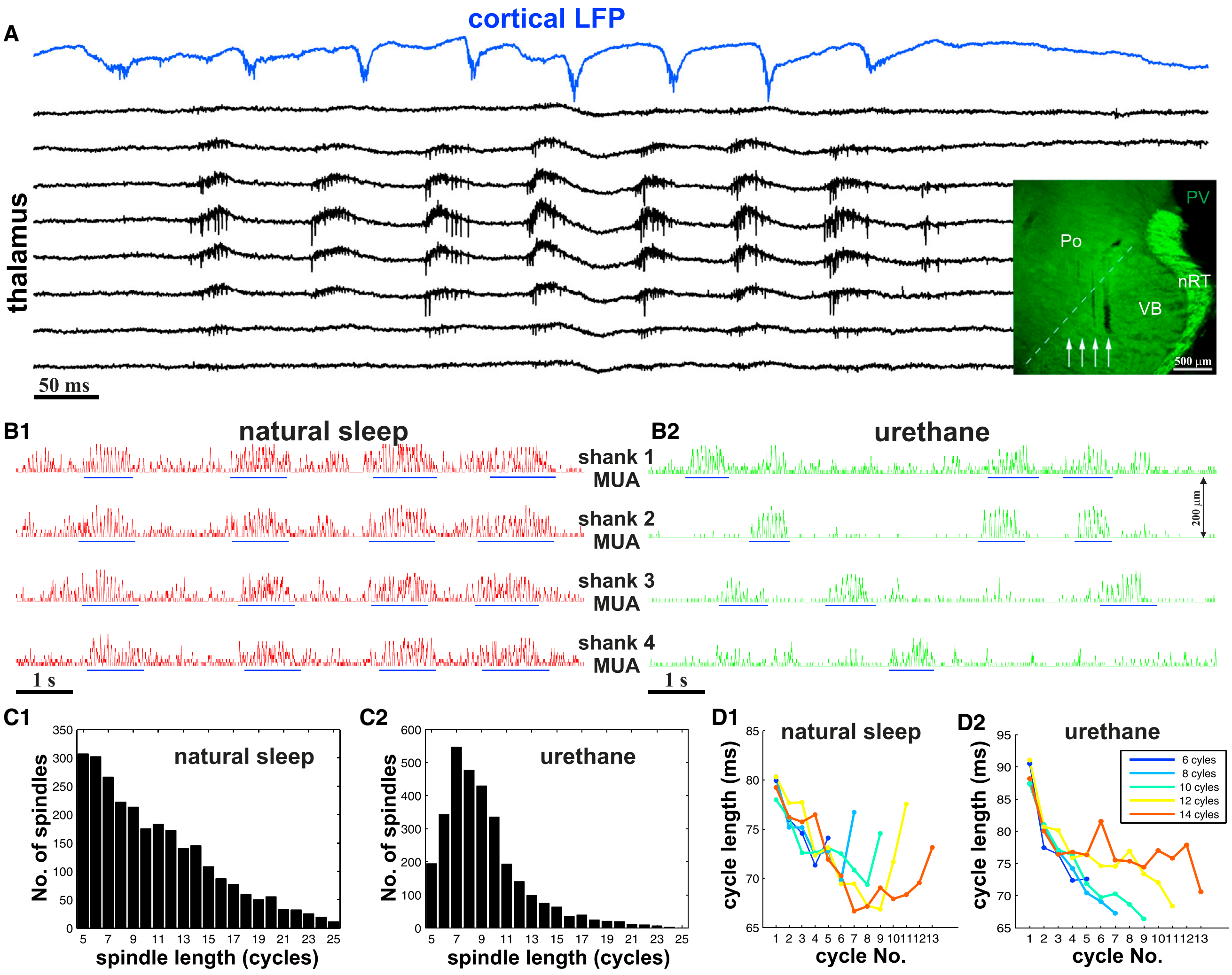

Figure 1. Sleep Spindles in Ventrobasal Thalamus

(A) Simultaneous cortical and thalamic recordings of a sleep spindle in a freely sleeping rat. The raw data show local field potentials in one cortical electrode and multiunit activity (MUA) in eight thalamic channels on one shank of a four shank electrode in the ventrobasal thalamus (VB) Inset: electrode tracks (white arrows) of the thalamic electrode. Note that all four tracks avoid nRT.

(B) Smoothed MUA recorded by each of the four electrode shanks (shank distance: 200 μm) in natural sleep (B1) and under urethane anesthesia (B2). Spindles (blue lines) appear as rhythmic elevations of MUA synchronously on all shanks in natural sleep but as a spatially restricted signal under urethane.

(C) Histogram of the spindle lengths from all unanesthetized (C1) and urethane anesthetized (C2) animals. Note the relative paucity of short spindles (five to six cycles) under urethane anesthesia.

(D) Changes in cycle length during spindles with different durations. Under natural sleep cycle lengths first increase, followed by a decrease before the end in all spindles (D1). Under urethane spindles accelerate throughout the spindle (D2).

spindles with different duration. We found that the synchrony of the two cell types remains unaltered during spindles, but nRT cells displayed robust duration specific activity. Optogenetic activation of spindles demonstrated that their duration is strongly constrained by the concurrent state of the thalamocortical network.

RESULTS

We performed multichannel silicon-probe recordings from the ventrobasal complex (VB) of urethane-anesthetized (n = 11) and naturally sleeping rats (n = 5) using silicon probes with four shanks, separated by 200 μm (Figure 1). Each shank was equipped with eight recording sites in an octrode configuration. In the majority of experiments, in addition to multiunit activity, a large number of single units were isolated by spike sorting (see below).

Sleep spindles were defined using thalamic multiunit recordings as an elevation of rhythmic multiunit firing above the background activity in the spindle frequency range (Figure 1A; see Experimental Procedures and Figure S1A available online). In naturally sleeping animals, sleep spindles (n = 3,190) appeared during slow-wave sleep as described before (Gaillard and Blois, 1981; Loomis et al., 1935; Silverstein and Levy, 1976; Steriade, 1999). Under urethane anesthesia, spindles (n = 2,975) were

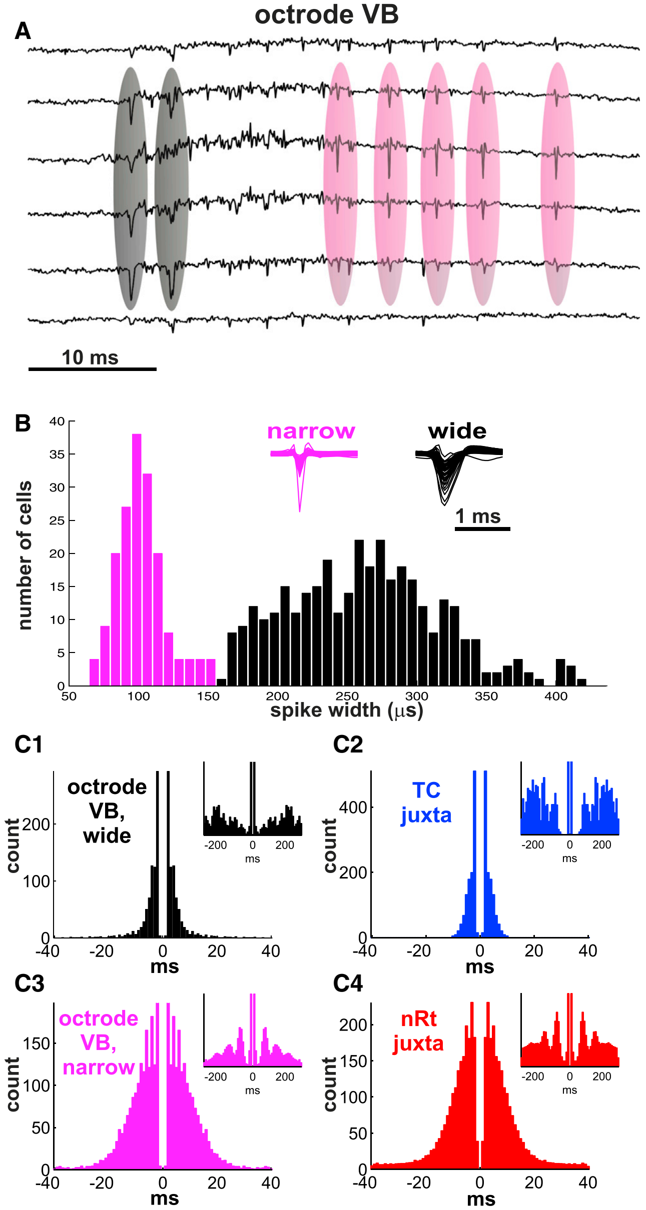

Figure 2. Two Types of Spike Waveforms in the Somatosensory Thalamus

(A) Raw data from six channels of a single octrode in VB showing wide (black circle) and narrow spikes (magenta circle).

(B) Bimodal distribution of action potential widths reveals two populations (black-wide spikes, magenta-narrow spikes).

(C) (C1 and C2) Autocorrelograms of a wide spike unit (C1) measured by silicon probe in VB compared to a TC cell (C2) recorded and labeled by juxtacellular recording in VB. (C3 and C4) The same two histogram for and a narrow spike unit (C3) recorded in VB, compared to a nRT cell (C4) measured by juxtacellular recording in nRT. Note wider base (longer burst) and spindle modulation (insets) in case of the narrow spike unit and the nRT cell. The figures are based on recordings made under urethane anesthesia.

present during the entire duration of the recordings albeit with variable rate of occurrence. In natural sleep, spindles were highly synchronous among the electrode shanks whereas under urethane the majority of spindles remained localized to one or two electrode shanks (Figure 1B). The mean spindle coherence between two shanks at 400 μm distance was 0.2 ± 0.06 for naturally sleeping and 0.09 ± 0.07 under urethane.

The mean duration of the spindles in both conditions agreed with previous reports (Azumi and Shirakawa, 1982; Gaillard and Blois, 1981; Silverstein and Levy, 1976) (10.7 ± 6.0 cycles/spindle in natural sleep, 9.5 ± 5.3 cycles/spindle under urethane). The number of short spindles (five to six cycles) was somewhat higher in natural sleep than under urethane (Figure 1C). The mean frequency of spindles was also similar in the two conditions (natural sleep 12.65 ± 1.89 Hz, urethane 12.91 ± 1.63 Hz). Both in natural sleep and under anesthesia, spindles showed an initially accelerating pattern, irrespective of their length (Figure 1D), as shown by Gardner et al. (2013). Spindles under natural sleep showed a deceleration toward the end, which was not present under urethane anesthesia.

Thus, we conclude that under our recording conditions sleep spindles can be reliable detected in the thalamus with comparable parameters (duration, frequency) to earlier results. The basic features of spindles under urethane and in freely sleeping conditions were largely similar, with the most prominent difference being that under anesthesia spindles were more spatially restricted.

Two Types of Spikes in Ventrobasal Thalamus

After spike sorting (see Experimental Procedures and Figure S1B), a single octrode yielded on average 12.9 well-separated single units (554 units all together from all animals). The action potential widths of single units clustered from VB showed a marked bimodality (Figures 2A and 2B), with the narrow-spike mode centered at 100 μs and a wide spike mode centered at 275 μs. The values of narrow spikes were actually briefer than the extracellular waveforms of cortical fast-spiking interneurons (Barthó et al., 2004). Units corresponding to both modes were usually recorded on a single shank. Wide-spike units (>150 μs) displayed burst firing typical of TC cells (Domich et al., 1986) (3.19 ± 1.52 spikes/burst, 149.0 ± 177.7 Hz for natural sleep, n = 102 units; 2.82 ± 1.11 spikes/burst, 287.0 ± 196 Hz under urethane, n = 320 units). Narrow-spike units (<150 μs) produced longer and slower bursts (5.17 ± 2.63 spikes/burst, 48.8 ± 81.5 Hz for natural sleep, n = 17 units, 3.57 ± 1.81 spikes/burst, 90.8 ± 119 Hz under urethane, n = 115 units) and were usually modulated in the spindle frequency range (Figure 2C). Cross-correlation analysis revealed that most narrow spike units fired on average 15–20 ms after wide spike units (Figure S1B4) both in natural sleep and under urethane anesthesia. These data suggested that beside TC cells (wide spikes) our electrodes sampled another neuronal population (narrow spikes). However, the origin of narrow spikes remained unclear because the rodent VB thalamus contains only one type of neuron, the TC cell (Barbaresi et al., 1986).

Narrow Spikes Belong to nRT Axon Terminals

The narrow spikes picked up by our electrodes in VB resembled axonal spikes that have been described in several neural systems (Goldberg and Fee, 2012; Khaliq and Raman, 2005; Meeks et al., 2005). Based on their waveform, bursting characteristics and the asymmetry of the cross-correlogramm with wide spikes, we

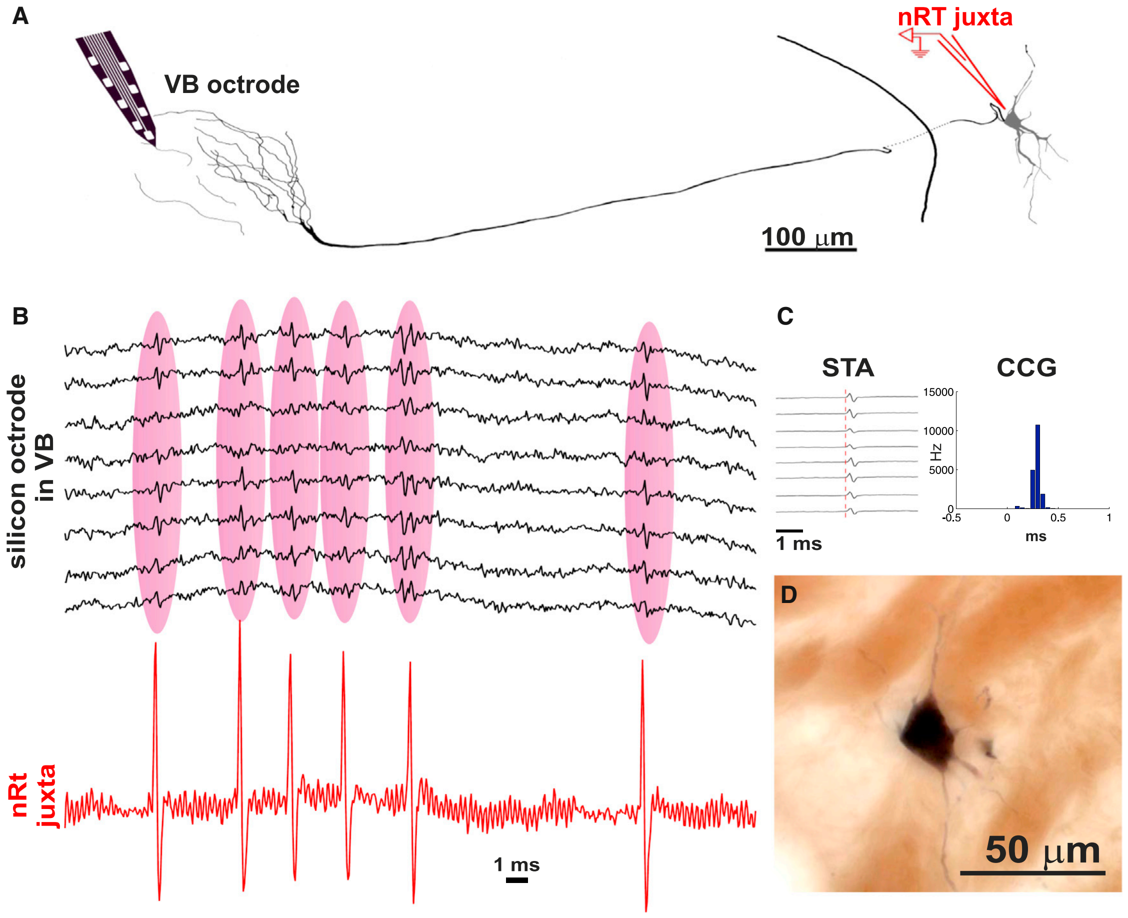

Figure 3. nRT Axonal Activity Recorded as Extracellular Signals in VB

(A) Camera lucida drawing of a juxtacellularly recorded and labeled nRT cell with simultaneous somatic recording in the nRT axonal recording in the VB. Note the axonal arbor around the octrode.

(B) Juxtacellular (bottom red) and silicon octrode (top black) traces of the recorded cell. Each somatic action potential is recorded as spikes in six out of the eight recording sites of the octrode, located 1 mm from the somatic electrode.

(C) Spike triggered averages (STA) and cross correlogram (CCG) of the octrode recordings triggered by the somatic nRT action potentials (red dashed line 0 ms).

(D) Photomicrograph of the recorded and labeled nRT cell.

hypothesized that narrow spikes represent axonal action potentials of nRT neurons. To test this, we first performed juxtacellular single unit recording and labeling from both the nRT (n = 21) and the VB (n = 10) under urethane anesthesia and compared the activity of identified cells with wide and narrow spikes. Under the same conditions, the activity of identified TC (Figure 2C2) and nRT (Figure 2C4) cells displayed identical features with wide (Figure 2C1) and narrow (Figure 2C3) spikes, respectively. Additionally, nRT neurons—like narrow spike units—showed pronounced spindle modulation (Figure 2C, insets).

To gain more direct evidence, we performed lesion experiments with the axon-sparing neurotoxin, kainic acid (KA) (n = 3). First, we selectively lesioned the TC cell bodies by iontophoresis of KA into VB, leaving the recording electrode in the same position. Before lesion, both wide and narrow spikes could be recorded in VB, whereas 4 hr after the lesion only narrow spikes remained in the same recording site (Figure S2). Spindle modulation of narrow spikes disappeared after VB lesion. When KA was injected into nRT, only narrow spikes were affected in VB.

Finally, in one case we were able to perform simultaneous somatic and axonal recording of the same nRT cell by combining silicon probe recording in VB with juxtacellular recording and labeling in nRT using neurobiotin-filled pipettes. The two electrodes were aligned according to the receptive field properties of the recorded units. Figure 3 shows a juxtacellularly recorded and labeled nRT neuron (Figure 3A), whose somatic action potentials were time-locked (<0.5 ms delay) to extracellular narrow spikes recorded in VB (Figures 3B and 3C). The silicon probe that recorded the narrow spikes was located approximately 1 mm caudomedially from the juxtacellular pipette. Morphological reconstruction of the juxtacellularly recorded neuron demonstrated a cell body located in nRT and axonal segments in close vicinity of the silicon probe (Figures 3A and 3D). Based on this direct evidence and the data listed above, we concluded that narrow spikes indeed represent axonal activity of nRT cells.

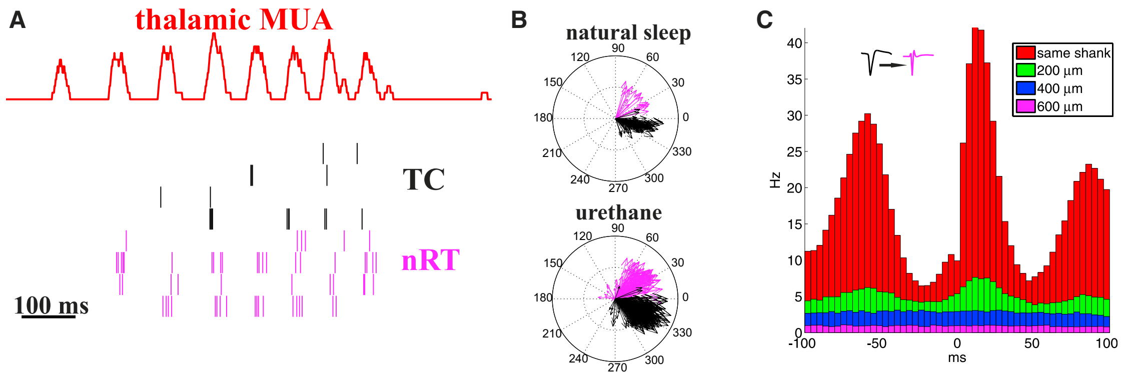

Figure 4. Local Interactions between TC and nRT Cells during Spindles

(A) A single spindle event displayed as smoothed multiunit activity in VB (red) together with TC cells (black) and nRT terminals (magenta) recorded by the same electrode shank under urethane anesthesia. The firing of both TC cells (black) and nRT axons (magenta) are locked to the local multiunit spindles. Note different firing frequency and burst length of the TC and nRT cells.

(B) Polar plots showing the phase vectors of individual TC and nRT cells recorded on the same shank relative to the multiunit spindle collected during several recordings. Top: natural sleep, bottom: urethane anesthesia. One spindle cycle is 360°. TC cells consistently fire at an earlier phase of the oscillation compared to nRT cells in both conditions.

(C) Cross correlograms between TC and nRT cells on the same electrode shank and between different shanks (200, 400, 600 μm apart) under urethane anesthesia. Robust correlation is evident only on the same shank (red), but modulation in the neighboring shank (green) also reaches significance. No correlation is apparent, however, on more distant shanks (blue, magenta).

We next asked whether the narrow spikes reflected nRT axon terminals, which synaptically interact with local TC cells, or passing axons, which do not. To do this, we took advantage of the localized nature of spindles under urethane. Connectivity between nRT and TC is strictly topographic and reciprocal, with a single nRT neuron typically restricting its entire axonal arbor to the same thalamic compartment it receives its major TC input from (Desîlets-Roy et al., 2002). The spatial scale of the axons arbor is typically of order 200 μm, similar to the shank separation distance of our multisite electrodes. Under urethane, the majority of spindles are restricted to one shank (Figure 1B). We therefore reasoned that if narrow spikes reflected the activity of nRT terminals, interactions between TC and nRT firing should occur within, but not between distant shanks during spindles. By contrast, if narrow spikes reflected passing axons, no significant correlation is expected because passing nRT axons cannot interact with TC cells. The data showed that both under urethane anesthesia and drug-free conditions, the activity of TC cells and nRT axon was not random. The two cell types fired phase-locked to the thalamic spindles within a shank at characteristically different phases (Figures 4A and 4B). When considering local spindles only (from urethane anesthetized recordings), cross-correlograms revealed strong correlation between TC cells and nRT axons recorded on the same shank (Figure 4C). This correlation was weaker at 200 μm and was not present between shanks 400 μm apart (Mann-Whitney test). Because the spatial extent of TC-nRT correlation was compatible with the size of nRT axon terminal arbor in VB (Pinault and Deschênes, 1998), we conclude that narrow spikes are generated by the axon terminals, not by passing fibers of nRT cells. The fact that axon terminals produced signals large enough to detect extracellularly, most probably resulted from the occurrence of strings of extremely closely spaced nRT boutons (Figure S3).

Constant Timing and Jitter during Spindles

Simultaneous recording of the somata of TC cells and the axon terminals of reciprocally connected nRT neurons allowed us to quantitatively investigate the structure of population activity during sleep spindles in a cycle-by-cycle basis in freely sleeping animals (Figures 5A and S4).

According to one hypothesis (see Introduction), spindles terminate due to disruption of thalamic synchrony by cortical input (Bonjean et al., 2011; Timofeev et al., 2001). This model predicts that the precision of TC-nRT interaction should be degraded as the spindle progresses. To test this, we computed cross-correlograms between the two cell populations for short (six cycles, n = 5,579) and long (14 cycles, n = 3,159) spindles for each consecutive cycles (Figure 5B). The cross-correlograms showed no marked difference in timing between spindles of different lengths and no change from cycle to cycle, indicating a constant latency of nRT activation by TC cells in every cycle of the spindles. We next assessed the jitter of TC-nRT synchrony by computing the SD of spike times relative to spindle peaks for every cycle in the same data set. This measure also showed no change with spindle progression (Figure 5C). Repeating the same two analyses for each cycle of every spindle length, in both freely sleeping and anesthetized animals, yielded identical results (Figure S5). None of the groups showed significant slope (Spearman's rank correlation $p > 0.1$). We therefore conclude that decreased TC-nRT efficacy and increased jitter among thalamic cells is not a major factor in spindle termination.

Decrease of nRT Activity before the Termination of Spindles

To study the alteration of TC and nRT activity during a spindle, we computed the cycle-by-cycle dynamics of excitatory and inhibitory activity for short (six cycles) and long (14 cycles)

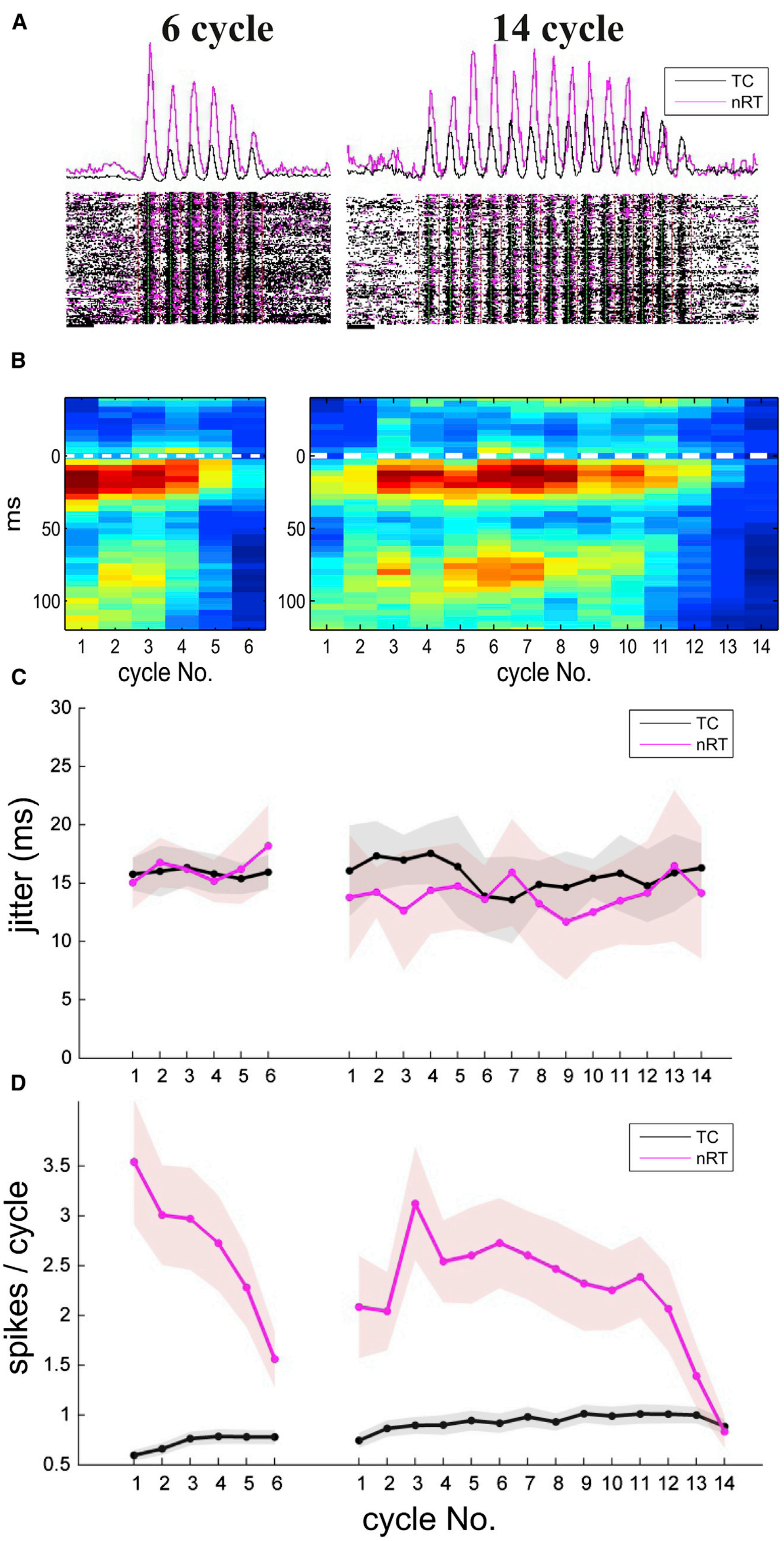

Figure 5. Cycle-by-Cycle Dynamics of Excitatory and Inhibitory Activity during Spindles of Different Lengths during Natural Sleep

(A) Perievent time histograms (top) and rasterplots (bottom) of TC (black) and nRT (magenta) units during spindles consisting of 6 (left) and 14 (right) cycles assembled from several representative sessions of three freely sleeping rats. Spindle peaks are aligned for better visibility.

(B) Cycle-by-cycle cross-correlograms of TC and nRT units shows unchanged peak latency during spindles of six and 14 cycles.

(C) Jitter (SD of spike distances from spindle peak) also remains stable during spindles. Each dot represents the mean data of a given cycle pooled across sessions and animals.

(D) Cycle-by-cycle alteration in the mean number of spikes per cycle for nRT (magenta) and TC cells (black) for short (six cycles, left) and long (14 cycles right) long. Note different trajectories of nRT but similar trajectories of TC cells. Shading indicates SEM.

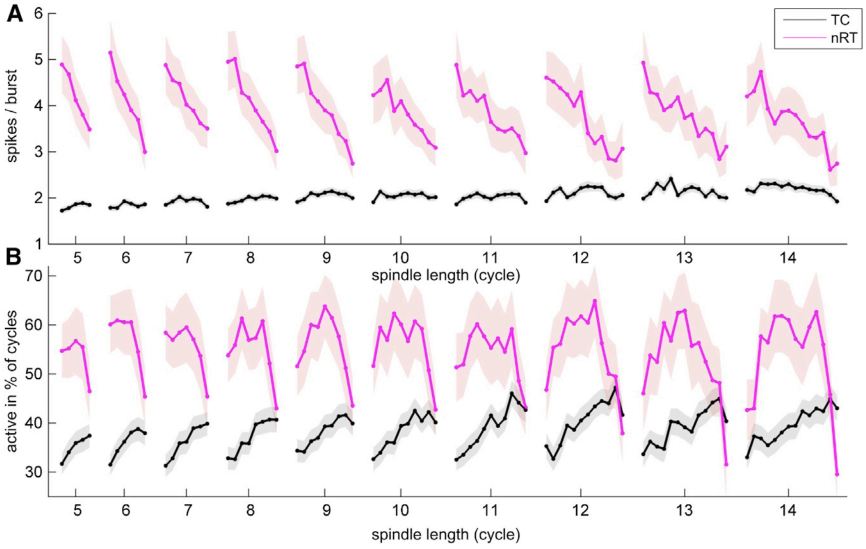

Figure 6. Probability of nRT Firing Displays Duration Specific Pattern during Natural Sleep

(A) Cycle-by-cycle changes in the mean number of spikes/bursts for TC (black) and nRT (magenta) cells during spindles of six to 14 cycles. nRT units display a steady decrease in spike per burst during spindles for all spindle lengths, whereas values of TC cells remain stable.

(B) Cycle-by-cycle changes in the probability of TC and nRT firing during spindles of different length. nRT cells display duration specific patterns. Each dot represents the mean data of a given cycle pooled across sessions and animals. Shading indicates SEM.

spindles (Figure 5D) in freely sleeping animals. During short spindles animals, nRT activity was highest in the first cycle (3.5 spikes/cycle) then decreased monotonically, dropping ~50% by the end of the spindle (1.55 spikes/cycle); by contrast, TC cell activity was lowest in the first cycle and increased steadily. For long spindles, nRT activity displayed a different, nonmonotonic pattern, first increasing from a moderate value (2.1 spikes/cycle) to reach a peak of 3.15 spikes/cycle by cycle 3 and then decreasing strongly to ~30% of the peak value (0.83 spikes/cycle) by spindle termination. During long spindles, TC activity again displayed a slow recruitment, in most cases with a slight decrease one to two cycles before the spindle ended.

Examining similar plots for spindles of all lengths (Figure S6A) indicated that in all cases nRT activity started to decrease several cycles before spindle termination, but this was not observed in case of TC cells in either natural sleep or urethane anesthesia. Based on these data, we conclude that nRT, but not TC activity starts to decay several cycles before the termination of all spindles.

Distinct nRT Activity Trajectories for Spindles with Different Length

The analysis above indicated that nRT cells may display spindle duration specific activity. To demonstrate this, we analyzed cycle-by-cycle TC and nRT activity for all spindle length. During spindles thalamic neurons fire exclusively in low-threshold Ca^{2+} bursts. Each neuron can produce one burst per spindle cycle but neither nRT nor TC cells fire at every cycle. As a consequence, changes in the number of spikes during consecutive cycles (as analyzed above) could reflect either a change in the number of spikes fired per burst, and/or a change in the probability the cell will fire a burst in the cycle (participation probability). It should be noted that participation probability is equivalent to the percentage of cells participating in a given spindle cycle, which indicates the level of recruitment within the TC or nRT population. To examine the cycle-by-cycle alterations in these measures, we calculated spike/burst and participation probability separately for all TC and nRT cells for all spindle length (five to 14 cycles) during natural sleep (Figure 6).

For nRT cells, the number of spikes per burst started at a uniformly high level (approximately five) for all spindle lengths and showed a monotonic decrease to approximately three to four spikes per burst by the end of the spindle. TC cells, on the other hand did not display significant alteration in burst size during the spindles (Figure 6A). For participation probability, nRT cells displayed pronounced differences between short and long spindles (Figure 6B). The shortest spindles were characterized by high initial nRT participation probability (60%), which dropped throughout the spindle to a moderate level (46%–49%) by termination. Longer spindles, however, started from a progressively lower probability levels (<40%) followed by an increase (reaching a plateau similar to the initial state of short spindles), then a decrease to a low level again. The endpoint of nRT participation probability was progressively lower with increasing spindle length. In contrast, the participation probability of TC cells displayed continuous increase during both long and short spindles until one to two cycles before spindle termination (from 35%–40% to 40%–45%). During natural sleep and under urethane anesthesia the spike per burst and probability trajectories were similar (Figure S6) confirming relatively intact spindle genesis under this anesthetic (for statistical analysis of the trajectories under both conditions see Figure S7).

We conclude that spindles are characterized by a progressive decrease in the burst size of nRT neurons. TC cells show a steady increase in participation probability irrespective of spindle length with no change in burst size. In addition nRT but not TC cells display distinct activity trajectories during short and long spindles.

Network State Controls Spindle Length

The large difference in duration-specific nRT activity prompted us to investigate how the measured variables at the first cycle correlate with the duration of spindles. The probability of nRT participation in the first cycle was strongly correlated with spindle duration ($r = -0.91$; $p < 0.001$; Figure 7A), whereas same measure of TC cells displayed only weakly significant correlation ($r = 0.63$; $p = 0.047$). In addition the number of spikes per burst in the first cycle also showed significant correlation with spindle

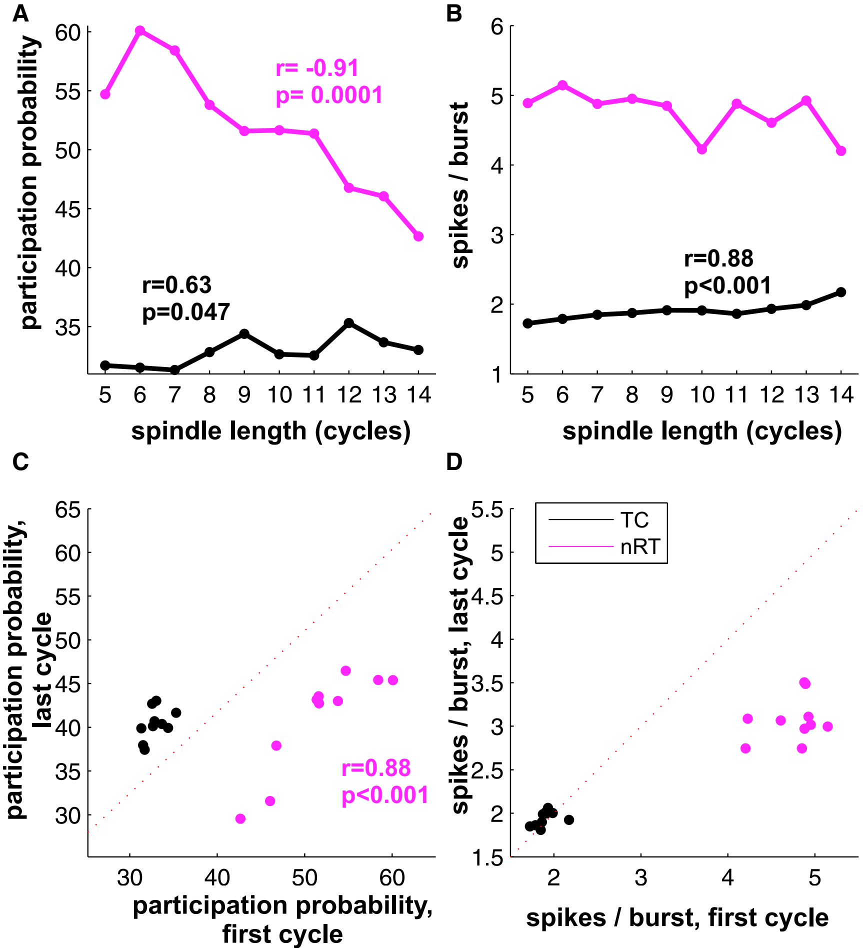

Figure 7. Initial Network State Correlates with Spindle Length during Natural Sleep

(A) Participation probability of nRT cells (magenta) in the in the first cycle strongly correlates with length of the spindle, TC cells (black) display weaker but still significant interaction.

(B) The initial number of spikes per burst in TC cells also correlates with the forthcoming spindle length.

(C) Correlation between the participation probability in the first and last cycle for TC (black dots) and nRT (magenta dots) cells. Between the initial and final state, only the nRT participation probability shows significant correlation.

(D) There is no correlation between the spikes/bursts in the initial and last cycle. In (A)–(D), each dot represents the mean value of spindles with given number of cycles pooled across sessions and animals. Only significant interactions are shown with numbers.

length in TC cells (Figure 7B). We also correlated the values of all variables between the first and last cycles. Only the probability of nRT participation between the first and last cycle displayed significant correlation ($r = 0.88$, $p < 0.001$; Figure 7C). Similar pattern was observed under urethane anesthesia (Figure S8). These data show that the length of the spindle was correlated with the pattern of neuronal activity measured on the first cycle and in case of nRT cells the activity follows a fixed trajectory to a well-determined endpoint.

These data allow two alternative scenarios about the control of spindle length. First, spindle length might be causally determined by nRT activity on the first cycle alone. Alternatively, the correlation might occur because first-cycle nRT activity is under the control of the ongoing network state.

To explore these possibilities we induced sleep spindles optogenetically (Halassa et al., 2011) in parvalbumin-channelrhodopsin (PV-ChR) (three animals, eight sessions) and vesicular-GABA transporter-channelrhodopsin (vGAT-ChR) mice (nine animals, 17 sessions). These strains express channelrhodopsin in both somata and axon terminals of nRT cells. Laser stimuli were delivered either to the nRT somata ($n = 10$), or to nRT axon terminals in VB ($n = 15$) with identical results. The experiments were performed under urethane anesthesia to gain large enough sample in a homogeneous state using the same multishank silicon probes as above. Under urethane anesthesia in mice, brain state showed cyclic fluctuations between patterns resembling slow-wave sleep, light sleep with sleep spindles (Figure 8A), and desynchronized EEG states, mimicking natural sleep on a shorter timescale (10–30 min). Spindles in mice had similar duration and frequency as in rats (12.9 ± 1.3 Hz, 914 ± 369 ms, n = 5,127 spindles).

Spindles were evoked by short stimuli of laser pulses with variable length and intensity (0.1–10 mW, 2–40 ms). Spindles could not be induced during desynchronized states or slow-wave activity, but only in the intermediate states in which spindles also occurred spontaneously (Figure 8A). During spindling epochs the length of both spontaneous and evoked spindles displayed large variability (Figure 8B), and there was a comodulation between the two ($R = 0.21$, $p < 0.001$). The density of spindles showed a weak correlation with the length of both spontaneous ($R = 0.09$, $p < 0.001$, 10 s window) and evoked spindles ($R = 0.11$, $p < 0.001$, 10 s window), indicating a slow background modulation. We found no significant correlation though, between the length of adjacent spindles.

We tested the effect of nRT population recruitment by varying either stimulus intensity ($n = 14$) or duration ($n = 11$) using stimulation parameters from subthreshold to maximal strength. The probability of evoking spindles increased both with stimulus intensity (Figure 8C, top), and duration (Figure 8D, top), ranging from 0% to 56%. This shows that the magnitude of nRT activation could be changed profoundly under these experimental conditions using the stimulus intensity range we applied. Still, in 20 out of 24 sessions, there was no correlation between stimulus intensity or duration and spindle length (Figures 8C and 8D, bottom; $p > 0.05$, Kruskal-Wallis test). The remaining four showed inconsistent and weak correlations in multiple directions. In four animals (six sessions), we kept the stimulus parameters and recording locations constant and summed the data across

animals. In this pooled data set also no significant difference was found between spindle length evoked by the three different stimulus intensities (0.14 mW, 4.4 mW, 10.5 mW, 1,200 repetitions each; Kruskal-Wallis test, p = 0.11). These results together indicate that the magnitude of of nRT cell activation does not directly correlate with spindle length. Rather, a constantly fluctuating network state controlls spindle duration probably via determining the size of recruitable nRT population.

Interestingly, the length distribution of spontaneous and evoked spindles differed significantly in 41.6% (10/24) of the experiments (Figure 8E; Mann-Whitney test), due to the absence of both the longest and shortest spindles in the evoked data. We suggest that these exceptional spindles arise from precisely calibrated population activity patterns that cannot be mimicked by laser stimulation.

DISCUSSION

We quantitatively characterized the dynamics of mutually connected excitatory TC and inhibitory nRT populations during sleep spindles in vivo. We found that nRT activity drops during the later phases of spindles irrespective of its length. In contrast, TC activity rose steadily throughout spindle duration. Activity trajectories in nRT cells, but not TC cells, were different between long and short spindles and the ongoing network activity strongly influenced spindle length.

Technical Considerations

The somatic activity of TC cells and the axonal activity of nRT cells were distinguished by nonoverlapping spike width, different firing and burst patterns, and different phase preference relative to the local spindle oscillation (Figures 2, 4, and S1B). Although extracellular axonal recordings of nRT cells have to our knowledge not been demonstrated before in freely sleeping animals, extracellular axonal recordings have previously been reported in other structures, and our spike width data are consistent with these earlier findings (Goldberg and Fee, 2012; Khaliq and Raman, 2005; Meeks et al., 2005; Robbins et al., 2013). In the present case, direct evidence for axonal recording has also been obtained by simultaneous recording of the soma and the axon of the same nRT cell (Figure 3). These anatomical and physiological data unambiguously demonstrate that when we measure TC somatic and nRT axonal activity via the same electrode shank we measure reciprocally coupled excitatory and inhibitory cell populations.

In every spindle cycle, TC cell activity preceded nRT activity by 15–20 ms (Figures 5B, S1B, and S5), followed by a longer delay (60–90 ms) before the next cycle started with the TC activity again. This pattern is fully consistent with the "ping-pong" mechanism of spindle genesis whereby TC firing induces an nRT burst, which in return evokes a prolonged inhibition in TC cells, enabling TC cells to fire a rebound burst and initiate the next cycle (von Krosigk et al., 1993).

The overall spindle dynamics were similar between natural sleep and urethane anesthesia, and the cycle-by-cycle trajectories of firing parameters in both TC and nRT cells displayed a surprisingly similar pattern (Figures S4, S5, and S6) despite the fact that urethane has been shown to have a depressing effect on neuronal excitability (Sceniak and Maciver, 2006). The most striking difference between freely sleeping and anesthetized spindles was in their spatial distribution: natural sleep was characterized by large-scale global spindle synchrony, whereas under urethane most spindles were restricted to a 200–400 μm volume (Figures 1B and 4C). Intriguingly, a similarly localized spindle pattern has been demonstrated in decorticated animals (Contreras et al., 1996, 1997). We therefore hypothesize that the localized nature of spindles under urethane anesthesia may reflect decreased corticothalamic activity relative to the naturally sleeping state.

Decrease of Inhibition and the Termination of Spindles

Three major theories have been put forward to explain the termination of spindles: that corticothalamic input desynchronizes the thalamic network during the waning of spindles (Bonjean et al., 2011); that progressive depolarization of TC cells unables them to fire rebound bursts toward the end of the spindle (Bal and McCormick, 1996; Lüthi and McCormick, 1998; Lüthi et al., 1998); or that spindles terminate due to progressive hyperpolarization of nRT cells (Bal et al., 1995b; Kim and McCormick, 1998). However, to date no cycle-by-cycle analysis of neuronal activity has been performed in freely sleeping animals.

Our data do not directly support the desynchronization hypothesis, because we did not find increased jitter before the termination of the spindles (Figures 5 and S5). Some aspects of our data are consistent with the TC cell depolarization hypothesis because the percentage of active TC cells progressively increased during each spindle. Nevertheless, we found no decrease in the number of TC spikes/burst toward the end of the spindles (Figures 5D, 6A, 6B, and S6), which would be expected if TC cells had become depolarized. Although recent data suggest that under the right conditions TC cells can still fire bursts even when depolarized, (Dreyfus et al., 2010), the fact that TC cells do not show reduced bursting at spindle termination argues against an exclusive role of TC depolarization in ending spindles.

The model of spindle termination most strongly supported by our data is instead progressive hyperpolarization of nRT cells (Bal et al., 1995a; Kim and McCormick, 1998). According to this hypothesis, inhibitory activity gradually decreases during the spindle, and once inhibitory input has decreased below a minimal value required for evoking rebound bursts in TC cells the oscillation will be terminated. Consistent with this possibility, we found that nRT burst size fell continuously throughout spindles of all durations, whereas the fraction of nRT cells active initially rose, before falling precipitously three to four cycles before spindle termination (Figures 5D, 6A, 6B, and S6). The mechanisms leading to the decreased nRT activity toward the end of the spindle remain to be established: whereas it may reflect conductances intrinsic to nRT neurons (Bal and McCormick, 1993; Cueni et al., 2008; Kim and McCormick, 1998), it could also result from alteration in corticothalamic input as suggested by Bonjean et al. (2011). Future modeling and experimental studies are thus required to elucidate the exact intracellular events underlying spindle termination.

Initial Network State and the Duration of Spindles

Two models can be put forward to control the duration of a transient neural oscillation. Length could be predetermined by the

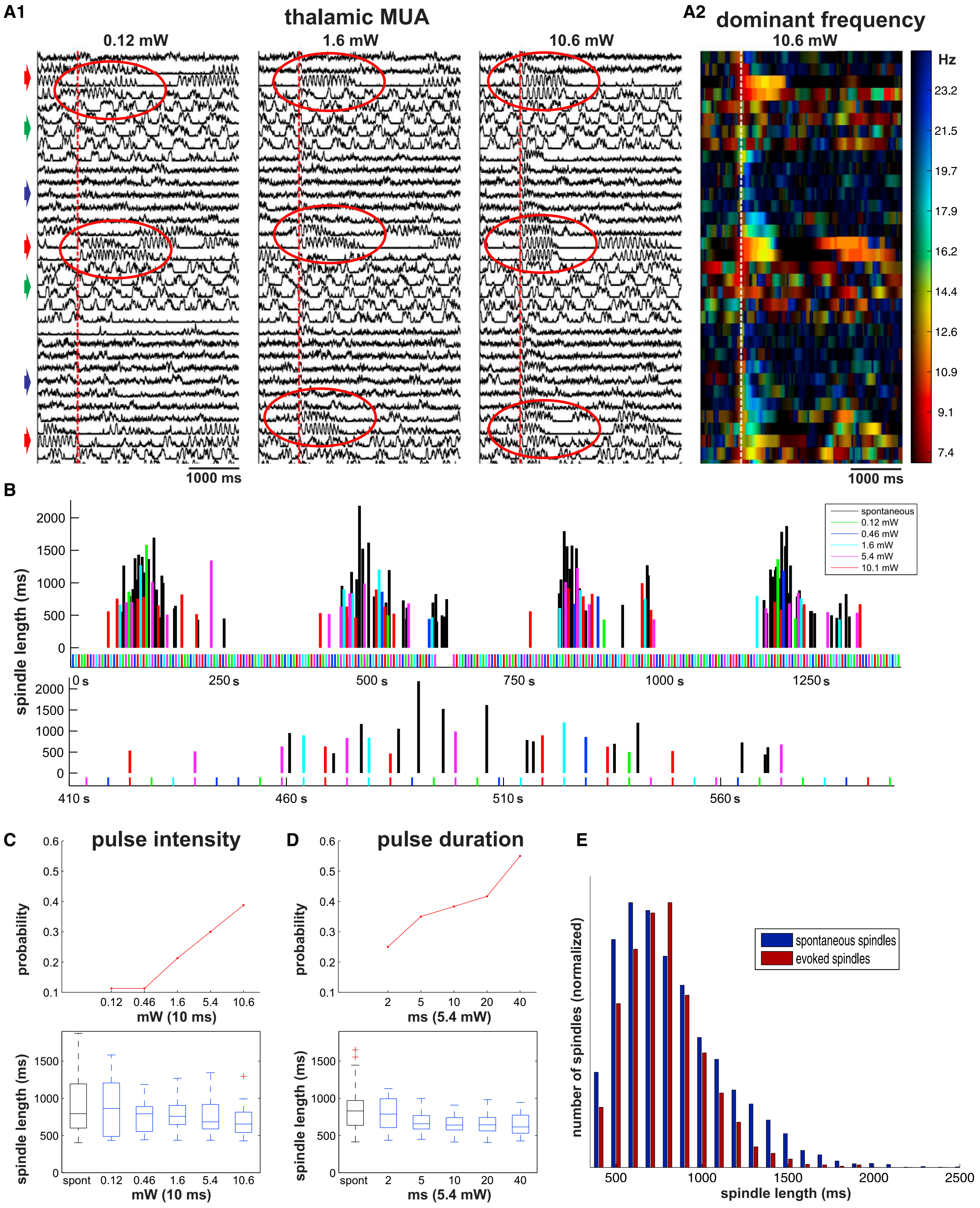

(legend on next page)

network state at the onset of the oscillation; alternatively, the oscillation could be stopped by a signal (extrinsic or intrinsic to the network) that emerges at a random time point once the transient is under way. In the first case, the oscillations are predicted to follow rigid activity trajectories, correlated with the initial state. In the latter case, no correlation is expected between initial state, end state, and duration.

Our data support the first hypothesis in the case of sleep spindles. We found a robust correlation between the participation probability of nRT cells in the first cycle and the length of the spindle (Figure 7A). A similar, though weaker relationship existed between spindle duration and both the participation probability and spike/burst of TC cells. We also observed a strong correlation between the participation probability of nRT cells in the first and the last cycles (Figure 7C). These data indicate that the initial state of the network has strong influence on spindle duration, and, once a spindle is launched, it does not evolve randomly but follows a rigid trajectory between fixed start and end points. The optogenetic experiments, however, indicated that there is no fixed correlation between the magnitude of nRT activation and the evoked spindle length. This suggests that spindle duration is determined by more complex variables, such as the precise state of neuromodulators and/or degree of cortical drive present at spindle initiation. Such variables would affect both the nRT firing pattern seen on the first cycle, and phenomena controlling spindle duration, such as the speed at which nRT cells become hyperpolarized as the spindle progresses.

Our data indicate that quantitative cycle-by-cycle analysis of excitatory and inhibitory activity can be used to test hypotheses regarding what determines the duration of transient network events. Because short, transient oscillations with widely different frequencies are abundant in the brain (e.g., type II theta activity, alpha waves, transient gamma oscillations, sharp wave ripples, etc.), similar analyses may help to determine the mechanisms of these oscillations. The duration of transient oscillatory events is plastic, changing both under healthy conditions (e.g., following learning) and also in case of neurological diseases. Thus, defining the mechanism underlying the duration of these transients can lead to better understanding of the temporal organization of neuronal activity in both healthy and diseased states.

EXPERIMENTAL PROCEDURES

Surgery and Recording

All animal procedures were approved by the Institute of Experimental Medicine Protection of Research Subjects Committee as well as the Food-Safety and Animal-Health Office of the Pest District Government Bureau, which is in line with the European Union regulation of animal experimentations. For general surgical procedures, see Barthó et al. (2004). Briefly, 41 male Wistar rats were used in the study. For anesthetized experiments (n = 36), rats were administered 1.5 g/kg urethane, the skull was opened over somatosensory cortex and thalamus (−3.0 AP, 2.8 ML from Bregma), dura was removed, and silicon microelectrodes (Neuronexus Technologies) were lowered into the brain. The probes used were mostly 32 site, four shank octrodes, in five thalamic experiments and most of cortical recordings 32 site linear probes.

In anesthetic-free, chronic experiments (n = 5), for the surgery the animals were anesthetized with a mixture (4 ml/kg) of ketamine (25 mg/ml), xylazine (1.3 mg/ml). Silicon probes were implanted above the thalamus attached to a custom-manufactured microdrive. After 1 week of recovery, the probes were moved gradually, and recordings were made at several depth locations. Tungsten wires (50 μm) were implanted to both primary somatosensory of motor cortices, also in hippocampus in three cases. Three of the five chronic animals yielded narrow spike units of clusterable quality.

For juxtacellular recording and labeling, glass micropipettes (20–70 MΩ) filled with 1.5% Neurobiotin (Vector Laboratories) were used. After perfusion, 60-μm-thick coronal brain sections were cut on a Vibratome and incubated with avidin-biotin-peroxidase complex (Vector Laboratories). The labeled cells were visualized using nickel-intensified diaminobenzidine (DAB) reaction. Labeled neurons and axonal trees were reconstructed using Camera Lucida. In case of dual nRT-VB recording experiments first a silicon probe was lowered into VB, and the receptive field of the multiunit was determined. Next, nRT units with a matching receptive field were then sought with several penetrations of a juxtacellular recording pipette.

Lesion experiments were performed by recording a baseline session from VB, followed by iotophoresis of 1% kainic acid (−2 μA, 7 s on/off cycle) for 20 min without moving the electrodes. Several (3–4) hours later postlesion session was recorded from the same electrode.

Optogenetics

Parvalbumin-channelrhodopsin and vesicular GABA-transporter-channelrhodopsin mouse strains were generated by crossing PV-cre or vGAT-cre (The Jackson Laboratory) mice with *-129S-Gt(ROSA)26Sortm32(CAG-COP4** *H134R/EYFP-Hze* (The Jackson Laboratory) reporter strains. For optical stimulation a 473 nm DPSS laser (LaserGlow) was used via a fiberport (Thorlabs) and a patch cord (Thorlabs) to the brain-implanted optic fiber. The optic fiber was either attached to the silicon probe in close proximity (<200 μm) of the recording site (axonal stimulation), or inserted directly into the nRT (somadendritic stimulation). Light intensity was modulated through the DPSS power supply, with a MATLAB-controlled DAQ-board (National Instruments). Stimulus strength was adjusted to span a range from near-threshold (~0.1 mW) to maximal effect (~10 mW).

Data Analysis

Extracellular signals were high-passed filtered (0.3 Hz), amplified (2,000 times) by a 64-channel amplifier, and digitized at 20 kHz with two National Instruments PCI-6259 cards. After detection, units were grouped by the semiautomatic "cluster cutting" algorithm ("KlustaKwik"; available at http://github.com/klusta-team) followed by manual clustering (Csicsvari et al., 2003).

Figure 8. Durations of Optogenetically Induced Spindles Do Not Correlate with Stimulus Intensity

(A) (A1) Vertically oriented traces of smoothed multiunit activity recorded by one electrode shank under urethane anesthesia in the VB of a mouse expressing channelrhodopsin-2 under parvalbumin promoter. Spindles were evoked by laser activation of nRT cells (red vertical lines) using three different laser intensities (0.12 mW, 1.6 mW, 10.6 mW) every 5 s. Note the state fluctuations between desynchronized (blue arrows), slow-wave sleep (green arrows), and lightly synchronized (red arrows) states with spontaneous spindles. Spindles can only be evoked (red ellipses) in the latter state. (A2) Dominant frequencies of the thalamic MUA activity on the rightmost traces in A1. Warm colors represent spindle frequencies.

(B) Duration of spontaneous (black) and evoked spindles (colored according to laser intensity) during a long recording. Evoked and spontaneous spindles co-occur in epochs. One of the epochs (blue dotted line) is shown in expanded time scale (bottom). The length of neighboring spindles show great variability.

(C) Probability of evoking a spindle increased with stimulus intensity (upper panel), but no significant difference (Kruskal-Wallis test) was found between the length of evoked spindles (lower panel).

(D) Same as (C), but, instead of stimulus intensity, stimulus duration was varied. Data in (A)–(D) are from the same animal.

(E) Distribution of all spontaneous and evoked spindle lengths summed from all animals and sessions. Note larger percentage of long (above 1,100 ms) and short (below 600 ms) spindles in spontaneous cases.

Auto- and cross-correlograms were inspected to verify the clustering procedure. The quality of spike clusters was estimated with the "isolation distance" measure (Schmitzer-Torbert et al., 2005) (Figure S1). Spike width was measured as the width of the extracellular spike waveform at half-amplitude (Barthó et al., 2004).

All data analysis was performed in MATLAB (MathWorks). Spindles were detected semiautomatically from the thalamic multiunit activity (MUA) separately for each shank (for details, see Figure S1). After automatic detection, spindles were verified visually, and false detections were deleted.

Spindle phases were estimated at the maximal amplitude of Morlet wavelet transform using scales between 7 and 20 Hz.

Jitter was defined as the SD of spike distances from spindle peak during a given cycle. For cycle-by-cycle cross-correlograms, only the reference spikes contained within the given cycle were considered. Number of spikes per burst in a cycle was estimated as the number of spikes fired, given the cell participated in a given cycle. Spike numbers per cycle, participation probability, and spikes per burst (Figures 5D, 6, and S6) were calculated for each spindle length category averaged across all cells in all animals.

Histological Analysis

Following the neurophysiological recordings, animals were transcardially perfused first with saline, and then with 400–500 ml of fixative containing 4% paraformaldehyde, 0.05% glutaraldehyde in 0.1 M phosphate buffer. Tissue blocks were cut on a Vibratome into 50 μm coronal sections. Electrode tracks were reconstructed from Nissl-stained slices (chronic experiments) or fluorescently counterstained for parvalbumin (acute experiments, the silicon probe was dipped in DII solution beforehand).

After lesion experiments, the fixed brain was cut into 50-μm-thick sections and or fluorescently counterstained for the neuronal marker NeuN to visualize the spread of lesion. The immunofluorescence stainings were performed according to the following protocol. Sections were intensively washed with PB and then treated with a blocking solution containing 5% normal goat serum (NGS) and 1% Triton-X for 45 min at room temperature. The primary antibody against PV (rabbit 1:3,000; Swant) and/or NeuN (mouse 1:300; Millipore) was diluted in PB containing 0.1% NGS and 0.2% Triton-X. After primary antibody incubation (overnight at room temperature), sections were treated with the secondary antibody Alexa-488-conjugated goat anti-rabbit or goat anti-mouse immunoglobulin (Ig)G and/or Alexa-594-conjugated goat anti-rabbit or goat anti-mouse IgG for 2 hr at room temperature. After further PB washes, sections were mounted in vectashield (Vector) and imaged using epifluorescent microscopy (Zeiss).

SUPPLEMENTAL INFORMATION

Supplemental Information includes eight figures and can be found with this article online at http://dx.doi.org/10.1016/j.neuron.2014.04.046.

ACKNOWLEDGMENTS

We thank Drs. G. Buzsáki, Z. Nusser, I. Soltész, J. Szabadics, and A. Lüthi for critical comments on the manuscript. The studies were supported by grants from the Hungarian Scientific Research Fund (OTKA NF101773, K109754 and K81357) the National Office for Research and Technology (NKTH-ANR, Neurogen), the Hungarian Brain Research Program - Grant No. KTIA_13_NAP-A-I/1 and KTIA_13_NAP-A-IV/1, EU FP7 NeuroSeeker, TÁMOP-4.2.1.B-11/2/KMR-2011-0002, Wellcome Trust (095668 and 094513), and EPSRC (EP/I005102). P.B. and F.M. are Bolyai fellows.

Accepted: April 17, 2014
Published: June 18, 2014

REFERENCES

Avanzini, G., Panzica, F., and de Curtis, M. (2000). The role of the thalamus in vigilance and epileptogenic mechanisms. Clin. Neurophysiol. *111* (*Suppl 2*), S19–S26.

Azumi, K., and Shirakawa, S. (1982). Characteristics of spindle activity and their use in evaluation of hypnotics. Sleep *5*, 95–105.

Bal, T., and McCormick, D.A. (1993). Mechanisms of oscillatory activity in guinea-pig nucleus reticularis thalami in vitro: a mammalian pacemaker. J. Physiol. *468*, 669–691.

Bal, T., and McCormick, D.A. (1996). What stops synchronized thalamocortical oscillations? Neuron *17*, 297–308.

Bal, T., von Krosigk, M., and McCormick, D.A. (1995a). Synaptic and membrane mechanisms underlying synchronized oscillations in the ferret lateral geniculate nucleus in vitro. J. Physiol. *483*, 641–663.

Bal, T., von Krosigk, M., and McCormick, D.A. (1995b). Role of the ferret perigeniculate nucleus in the generation of synchronized oscillations in vitro. J. Physiol. *483*, 665–685.

Barbaresi, P., Spreafico, R., Frassoni, C., and Rustioni, A. (1986). GABAergic neurons are present in the dorsal column nuclei but not in the ventroposterior complex of rats. Brain Res. *382*, 305–326.

Barthó, P., Hirase, H., Monconduit, L., Zugaro, M., Harris, K.D., and Buzsáki, G. (2004). Characterization of neocortical principal cells and interneurons by network interactions and extracellular features. J. Neurophysiol. *92*, 600–608.

Bódizs, R., Kis, T., Lázár, A.S., Havrán, L., Rigó, P., Clemens, Z., and Halász, P. (2005). Prediction of general mental ability based on neural oscillation measures of sleep. J. Sleep Res. *14*, 285–292.

Bonjean, M., Baker, T., Lemieux, M., Timofeev, I., Sejnowski, T., and Bazhenov, M. (2011). Corticothalamic feedback controls sleep spindle duration in vivo. J. Neurosci. *31*, 9124–9134.

Buzsáki, G. (2006). Rhythms of the Brain. (New York: Oxford University Press).

Contreras, D., Destexhe, A., Sejnowski, T.J., and Steriade, M. (1996). Control of spatiotemporal coherence of a thalamic oscillation by corticothalamic feedback. Science *274*, 771–774.

Contreras, D., Destexhe, A., and Steriade, M. (1997). Spindle oscillations during cortical spreading depression in naturally sleeping cats. Neuroscience *77*, 933–936.

Csicsvari, J., Henze, D.A., Jamieson, B., Harris, K.D., Sirota, A., Barthó, P., Wise, K.D., and Buzsáki, G. (2003). Massively parallel recording of unit and local field potentials with silicon-based electrodes. J. Neurophysiol. *90*, 1314–1323.

Cueni, L., Canepari, M., Luján, R., Emmenegger, Y., Watanabe, M., Bond, C.T., Franken, P., Adelman, J.P., and Lüthi, A. (2008). T-type Ca2+ channels, SK2 channels and SERCAs gate sleep-related oscillations in thalamic dendrites. Nat. Neurosci. *11*, 683–692.

Desîlets-Roy, B., Varga, C., Lavallée, P., and Deschênes, M. (2002). Substrate for cross-talk inhibition between thalamic barreloids. J. Neurosci. *22*, RC218.

Domich, L., Oakson, G., and Steriade, M. (1986). Thalamic burst patterns in the naturally sleeping cat: a comparison between cortically projecting and reticularis neurones. J. Physiol. *379*, 429–449.

Dreyfus, F.M., Tscherter, A., Errington, A.C., Renger, J.J., Shin, H.-S., Uebele, V.N., Crunelli, V., Lambert, R.C., and Leresche, N. (2010). Selective T-type calcium channel block in thalamic neurons reveals channel redundancy and physiological impact of I(T)window. J. Neurosci. *30*, 99–109.

Ferrarelli, F., Huber, R., Peterson, M.J., Massimini, M., Murphy, M., Riedner, B.A., Watson, A., Bria, P., and Tononi, G. (2007). Reduced sleep spindle activity in schizophrenia patients. Am. J. Psychiatry *164*, 483–492.

Fogel, S.M., Smith, C.T., and Cote, K.A. (2007). Dissociable learning-dependent changes in REM and non-REM sleep in declarative and procedural memory systems. Behav. Brain Res. *180*, 48–61.

Gaillard, J.M., and Blois, R. (1981). Spindle density in sleep of normal subjects. Sleep *4*, 385–391.

Gardner, R.J., Hughes, S.W., and Jones, M.W. (2013). Differential spike timing and phase dynamics of reticular thalamic and prefrontal cortical neuronal populations during sleep spindles. J. Neurosci. *33*, 18469–18480.

Gibbs, E.L., and Gibbs, F.A. (1962). Extreme spindles: correlation of electroencephalographic sleep pattern with mental retardation. Science *138*, 1106–1107.

Goldberg, J.H., and Fee, M.S. (2012). A cortical motor nucleus drives the basal ganglia-recipient thalamus in singing birds. Nat. Neurosci. *15*, 620–627.

Halassa, M.M., Siegle, J.H., Ritt, J.T., Ting, J.T., Feng, G., and Moore, C.I. (2011). Selective optical drive of thalamic reticular nucleus generates thalamic bursts and cortical spindles. Nat. Neurosci. *14*, 1118–1120.

Huguenard, J.R., and McCormick, D.A. (2007). Thalamic synchrony and dynamic regulation of global forebrain oscillations. Trends Neurosci. *30*, 350–356.

Khaliq, Z.M., and Raman, I.M. (2005). Axonal propagation of simple and complex spikes in cerebellar Purkinje neurons. J. Neurosci. *25*, 454–463.

Kim, U., and McCormick, D.A. (1998). Functional and ionic properties of a slow afterhyperpolarization in ferret perigeniculate neurons in vitro. J. Neurophysiol. *80*, 1222–1235.

Kostopoulos, G.K. (2000). Spike-and-wave discharges of absence seizures as a transformation of sleep spindles: the continuing development of a hypothesis. Clin. Neurophysiol. *111* (*Suppl 2*), S27–S38.

Loomis, A.L., Harvey, E.N., and Hobart, G. (1935). Potential rhythms of the cerebral cortex during sleep. Science *81*, 597–598.

Lüthi, A., and McCormick, D.A. (1998). Periodicity of thalamic synchronized oscillations: the role of Ca2+-mediated upregulation of Ih. Neuron *20*, 553–563.

Lüthi, A., Bal, T., and McCormick, D.A. (1998). Periodicity of thalamic spindle waves is abolished by ZD7288,a blocker of Ih. J. Neurophysiol. *79*, 3284–3289.

Meeks, J.P., Jiang, X., and Mennerick, S. (2005). Action potential fidelity during normal and epileptiform activity in paired soma-axon recordings from rat hippocampus. J. Physiol. *566*, 425–441.

Morin, A., Doyon, J., Dostie, V., Barakat, M., Hadj Tahar, A., Korman, M., Benali, H., Karni, A., Ungerleider, L.G., and Carrier, J. (2008). Motor sequence learning increases sleep spindles and fast frequencies in post-training sleep. Sleep *31*, 1149–1156.

Nicolas, A., Petit, D., Rompré, S., and Montplaisir, J. (2001). Sleep spindle characteristics in healthy subjects of different age groups. Clin. Neurophysiol. *112*, 521–527.

Picard, F., Mégevand, P., Minotti, L., Kahane, P., Ryvlin, P., Seeck, M., Michel, C.M., and Lantz, G. (2007). Intracerebral recordings of nocturnal hyperkinetic seizures: demonstration of a longer duration of the pre-seizure sleep spindle. Clin. Neurophysiol. *118*, 928–939.

Pinault, D., and Deschênes, M. (1998). Projection and innervation patterns of individual thalamic reticular axons in the thalamus of the adult rat: a three-dimensional, graphic, and morphometric analysis. J. Comp. Neurol. *391*, 180–203.

Robbins, A.A., Fox, S.E., Holmes, G.L., Scott, R.C., and Barry, J.M. (2013). Short duration waveforms recorded extracellularly from freely moving rats are representative of axonal activity. Front. Neural Circuits *7*, 181.

Sceniak, M.P., and Maciver, M.B. (2006). Cellular actions of urethane on rat visual cortical neurons in vitro. J. Neurophysiol. *95*, 3865–3874.

Schmitzer-Torbert, N., Jackson, J., Henze, D., Harris, K., and Redish, A.D. (2005). Quantitative measures of cluster quality for use in extracellular recordings. Neuroscience *131*, 1–11.

Shibagaki, M., Kiyono, S., and Watanabe, K. (1982). Spindle evolution in normal and mentally retarded children: a review. Sleep *5*, 47–57.

Silverstein, L.D., and Levy, C.M. (1976). The stability of the sigma sleep spindle. Electroencephalogr. Clin. Neurophysiol. *40*, 666–670.

Steriade, M. (1999). Coherent oscillations and short-term plasticity in corticothalamic networks. Trends Neurosci. *22*, 337–345.

Steriade, M., and Deschenes, M. (1984). The thalamus as a neuronal oscillator. Brain Res. *320*, 1–63.

Steriade, M., Deschênes, M., Domich, L., and Mulle, C. (1985). Abolition of spindle oscillations in thalamic neurons disconnected from nucleus reticularis thalami. J. Neurophysiol. *54*, 1473–1497.

Timofeev, I., Bazhenow, M., Sejnowski, T., and Steriade, M. (2001). Contribution of intrinsic and synaptic factors in the desynchronization of thalamic oscillatory activity. Thal. Rel. Syst. *1*, 53–69.

von Krosigk, M., Bal, T., and McCormick, D.A. (1993). Cellular mechanisms of a synchronized oscillation in the thalamus. Science *261*, 361–364.

Neuron, Volume *82*

Supplemental Information

Ongoing Network State Controls the Length of Sleep Spindles via Inhibitory Activity

Péter Barthó, Andrea Slézia, Ferenc Mátyás, Lejla Faradzs-Zade, István Ulbert, Kenneth D. Harris, and László Acsády

Supplementary Figures

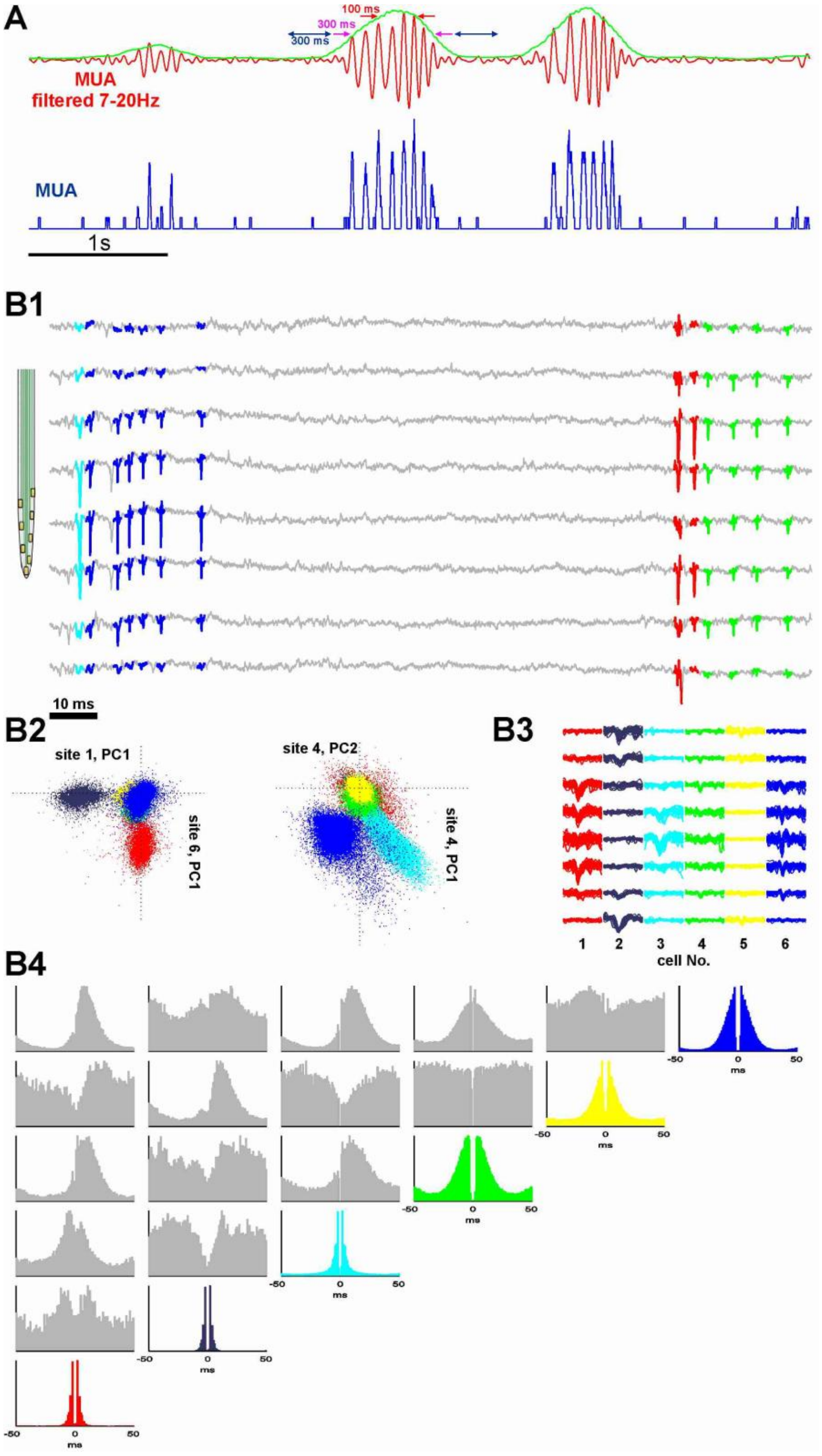

Supplementary Figure 1, related to Figure 1. Spindle detection and unit clustering

A) Detection of multiunit spindles in the thalamus. Blue trace, local thalamic multiunit activity smoothed with a 11 ms long square kernel; red trace, the same trace filtered between 7-20 Hz; green trace, envelope fitted on red trace, used for spindle detection. Criteria for spindles were: the envelope should exceed 1.5 standard deviations of fMUA for >300ms (magenta arrow), with a central portion of the envelope exceeding 2.5*SD for >100 ms (red arrow), and a relative silence below 1.5 STD for 300 ms before and after the spindle (blue arrow). **B**) Overview of the spike clustering procedure**.** (**B1**) Raw trace from VB recorded by the eight recording sites of on one shank (left) of the four-shank octrode. Spike waveforms of four different neurons can be distinguished during the period shown. (**B2**) Two-dimensional projections of the clustered action potentials from the same shank. (**B3**) Spike waveforms of the clustered neurons. (**B4**) Auto- and crosscorrelograms of the same cells. Note the different shapes of the autocorrelogram in case of units 1-2-3 vs. 4-5-6, and the asymmetric cross-correlation between the two groups.

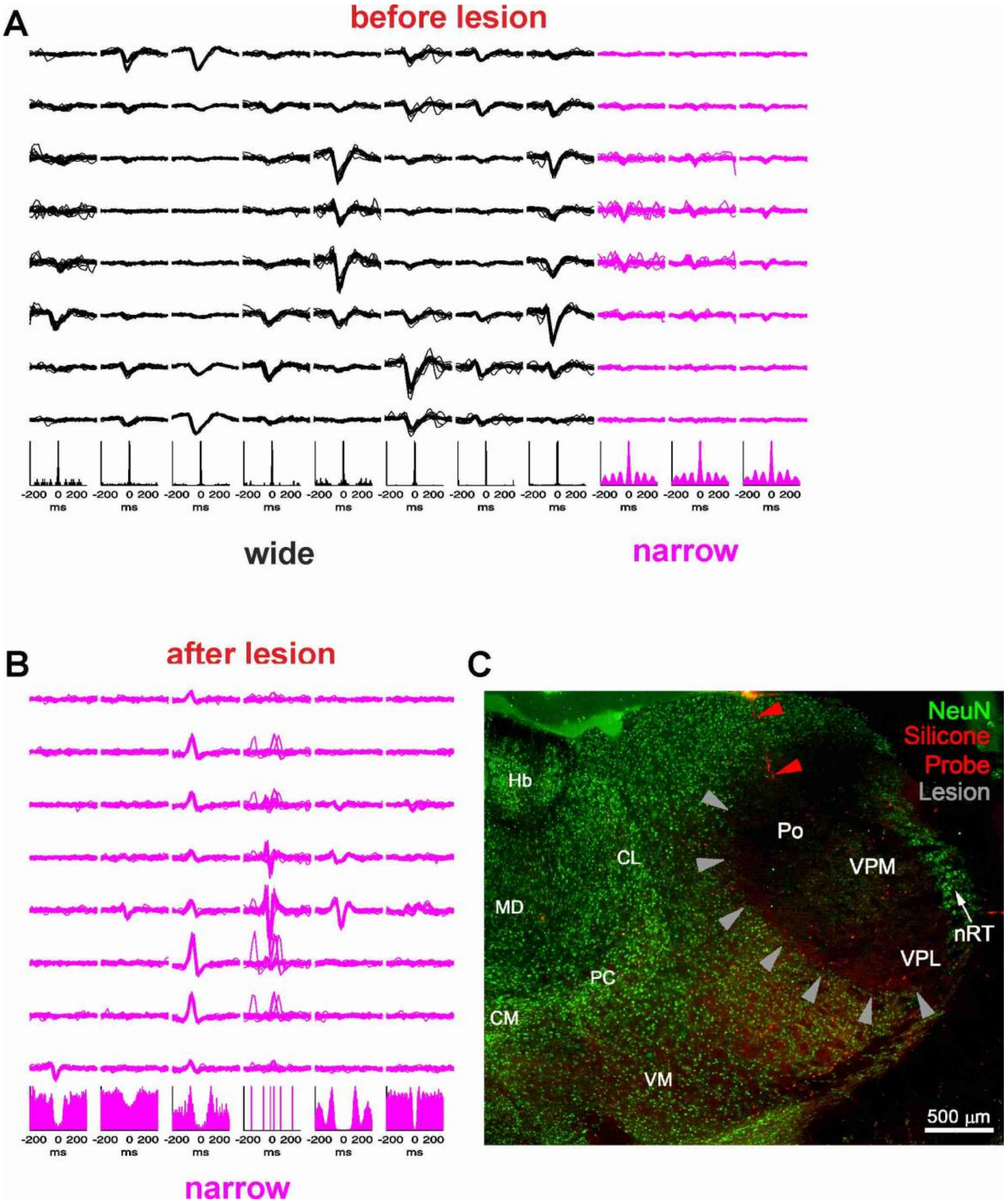

Supplementary Figure 2, related to Figure 3. Wide spikes selectively disappear from VB after focal kainic acid lesion.

(**A**) Spike waveforms on the eight recording sites of wide spikes (black, 8 units) and narrow spikes (magenta, 3 units) recorded by one of the shanks inserted into the VB before the lesion. The autocorrelograms of each unit are shown at the bottom of the columns. Note pronounced modulation of narrow spikes at the spindle frequency range. (**B**) Four hours after the KA lesion only narrow spike units can be recorded at the same location (silicon probe not moved). Spindle modulation disappears from the autocorrelograms (**C**) NeuN staining shows complete absence of thalamocortical cells (grey arrowheads) after iontophoresis of kainic acid into VB. Red arrowheads; electrode track.

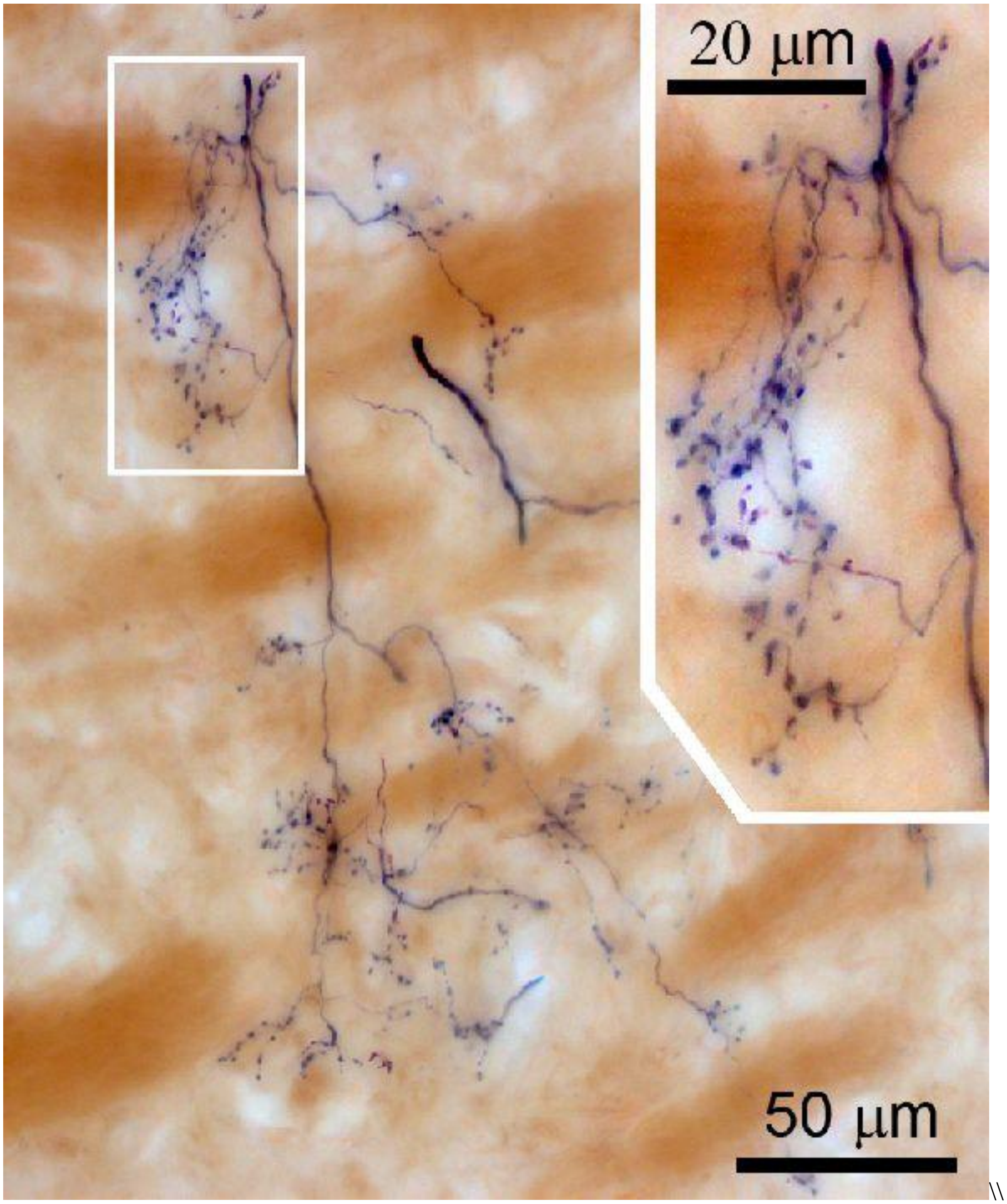

\\

Supplementary Figure 3, related to Figure 3. Dense axon terminal clusters of nRT cells in VB. Part of the axon terminal arbor of a juxtacellularly filled nRT neuron in VB. Note the extremely dense clusters of axon terminals. The right inset shows that within a reasonably small volume of neuropyl (>20 micron) close to 100 terminals of the same axon branch can be present. Since this terminal cluster fire nearly simultaneously, we predict that it generates a large enough extracellular field response which can be recorded by several recording sites of the same electrode shank.

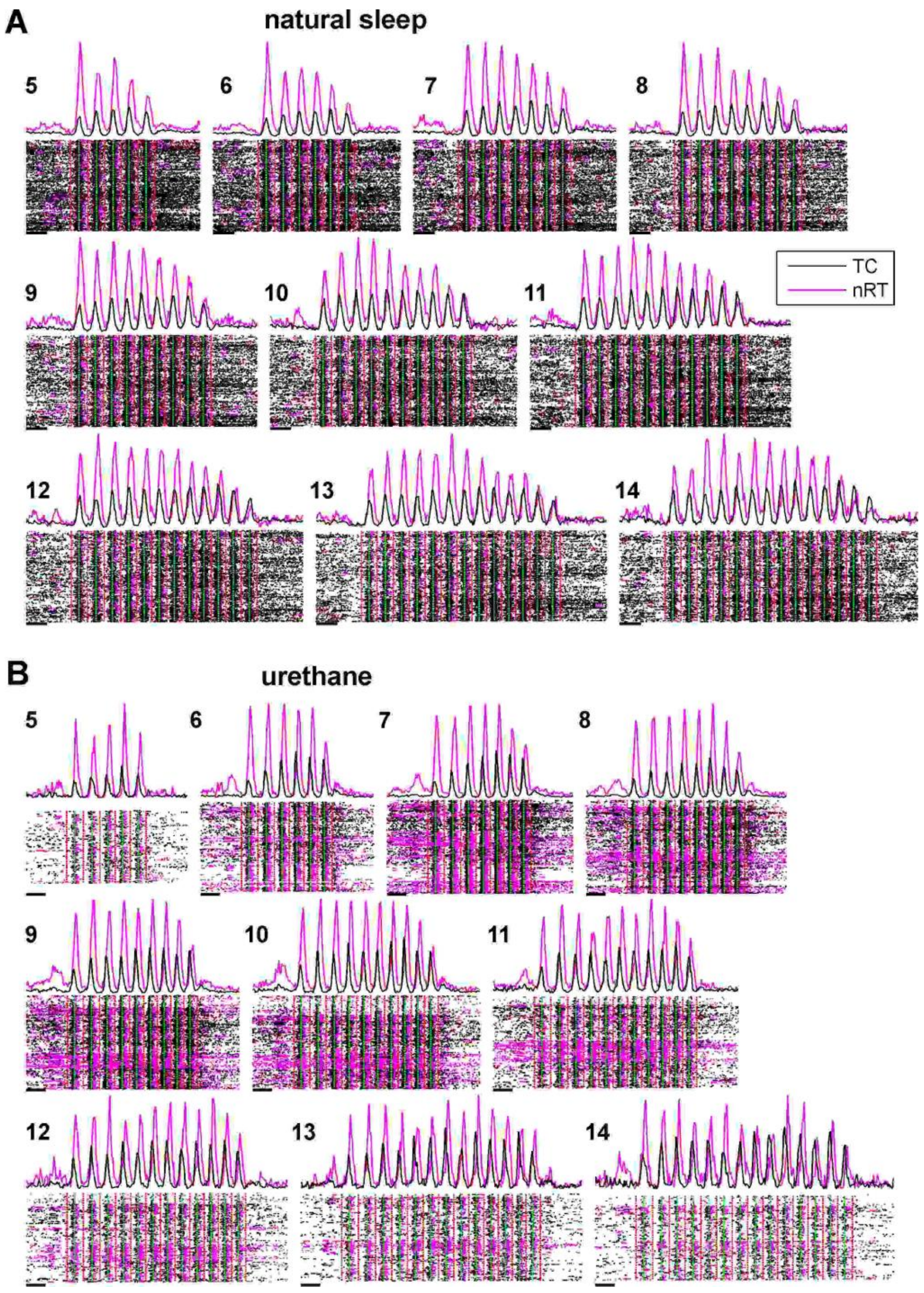

Supplementary Figure 4, related to Figure 5. Network activity of TC and nRT cells during natural sleep and urethane anesthesia.

Perievent time histograms (PSTH) and rasterplots of TC (black) and nRT (magenta) activity during spindles for all lengths under natural sleep (**A**), and urethane anesthesia (**B**). PSTHs are aligned to the peak of the cycles for visualisation purposes (here and Fig3A only). The plots are arranged from short spindles (5 cycles top left) to long spindles (14 cycles) bottom right. The plots display recordings from several animals and recording locations. Note that in both conditions short spindles start with high nRT actvity which decreases cycle to cycle. This scenario changes progressively to lower initial nRT activity with increasing spindle length. Scales: 100ms

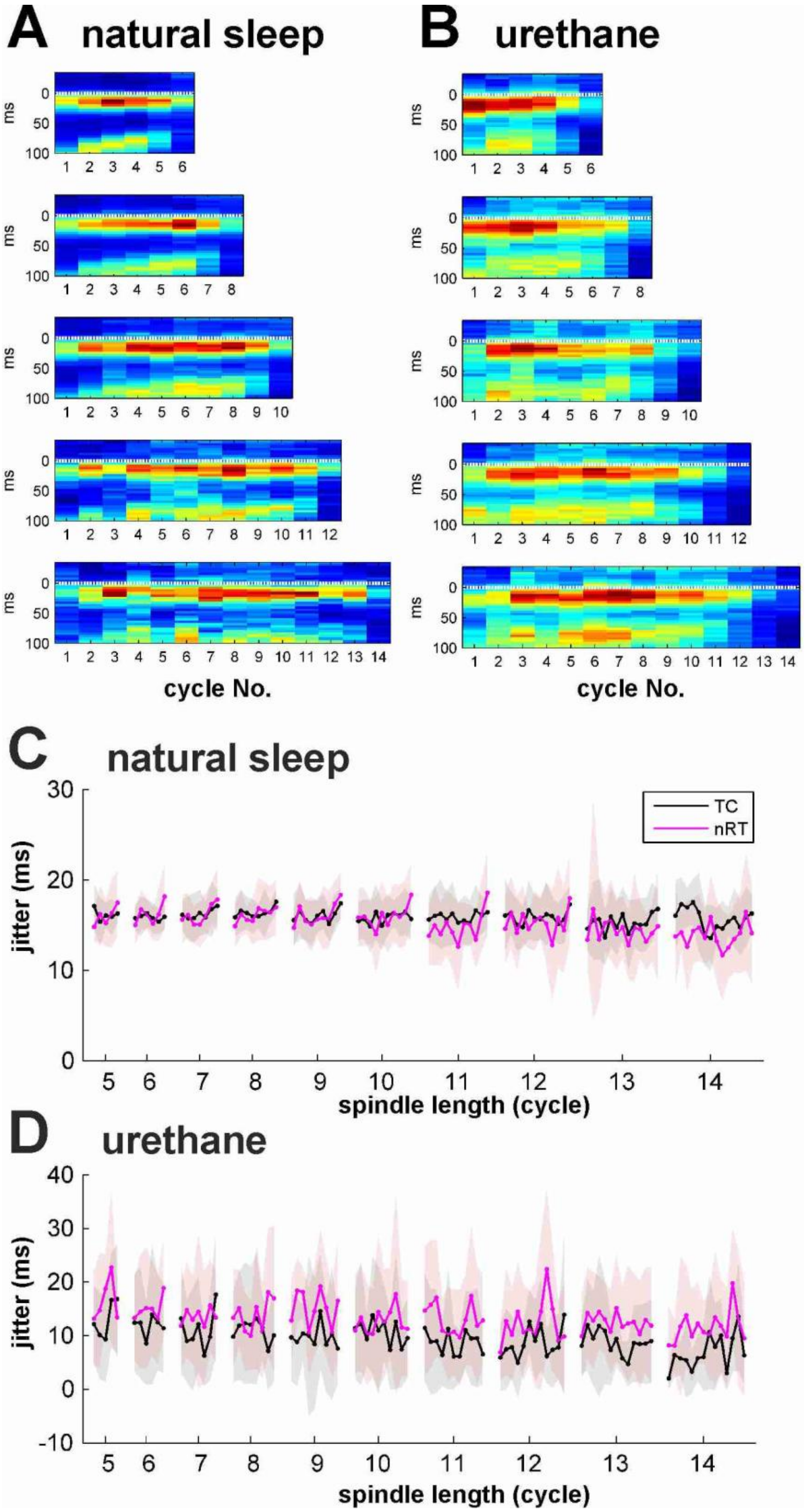

Supplementary Figure 5, related to Figure 5. Stable cross correlation and jitter of TC and nRT units.

A-B) Summed cycle-by-cycle cross-correlograms between TC and nRT cells for several (6,8,10,12,14 cycles) spindle lengths in naturally sleeping (**a**), and urethane anesthetized (**b**) animals. Warm colors indicate higher values. TC cells always precede nRT with a stable delay despite the changes in the number of spikes fired. **C-D)** Cycle-by-cycle change in jitter measured as SD of action potential distance from peak in TC and nRT cells for all spindle lengths in naturally sleeping (**A**), and urethane anesthetized (**B**) animals. Shading indicates SEM.

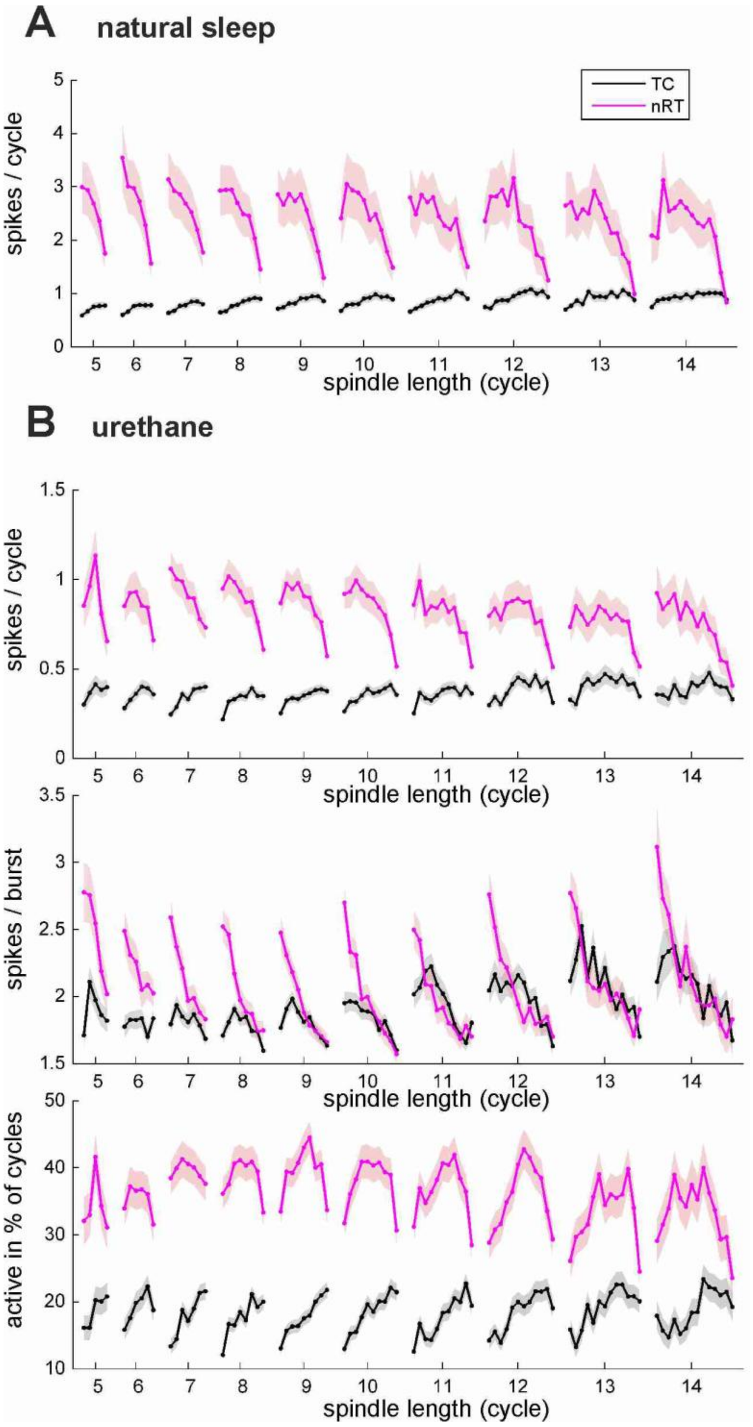

Supplementary Figure 6, related to Figures 5,6. Cycle-by-cycle neuronal activity during spindles of natural sleep and under urethane anesthesia.

(**A**) Mean number of spikes per cycle for all spindle lengths under natural sleep for TC cells (black), nRT cells (magenta) and the ratio of the two activities (red). Note the increasing ratio of excitatory / inhibitory actvity throughout the spindles (**B**) Mean number of spikes per cycle, number of spikes per burst, and firing probability for all spindle lengths under urethane anesthesia. Compare to (**A**) and Figure 4. Shading indicates SEM.

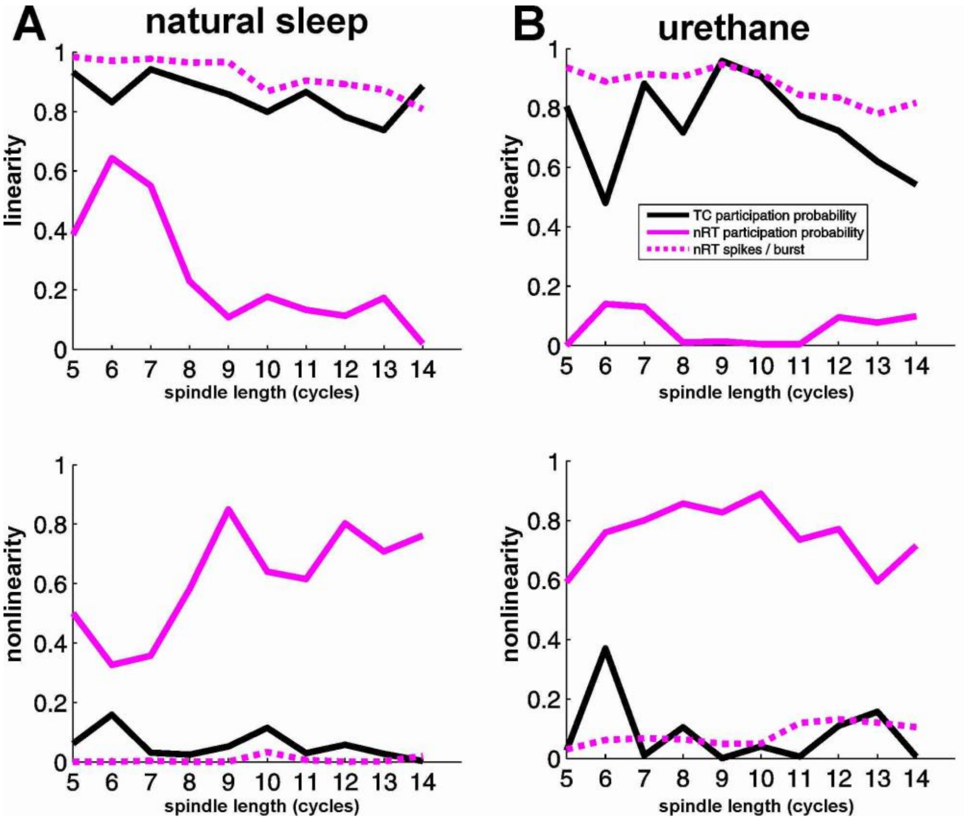

Supplementary Figure 7, related to Figure 6.. Shape analysis of trajectories
(**A**) Linearity and nonlinearity of curves for all spindle lengths on Figs. 6 and S8. Linearity was defined as R^2_{linear}, nonlinearity as R^2_{linear} - $R^2_{quadratic}$, for linear and quadratic fits of the curve, respectively. TC participation probability and nRT spikes/burst are linear in all groups, while nRT participation probability is more linear for short spindles (<8 cycles), and more nonlinear for long spindles. TC spikes/burst was omitted for having too low variance explained. (**B**) Same curves under urethane anesthesia. Note that nRT participation probability is nonlinear even for short spindles.

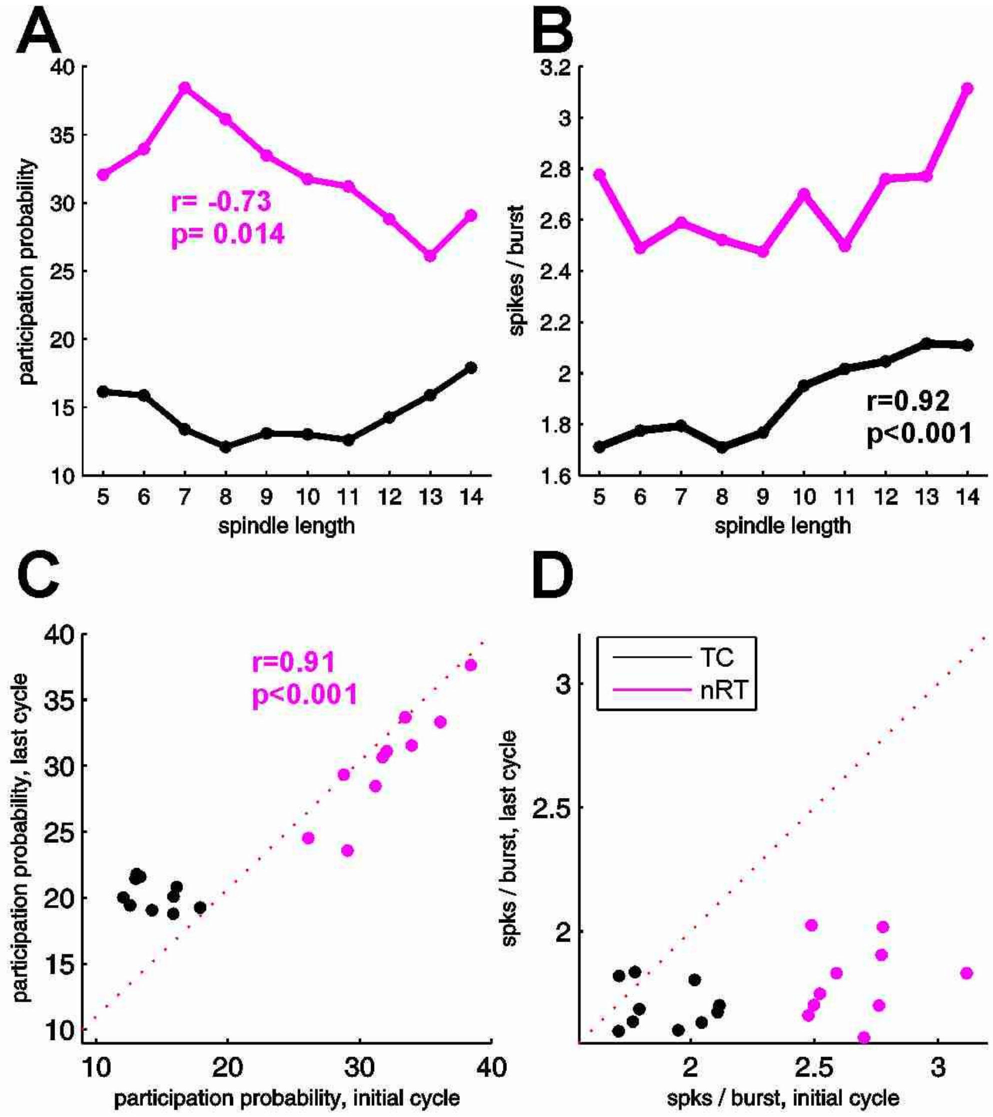

Supplementary Figure 8, related to Figure 7. Initial network state correlates with spindle length under urethane anesthesia

(**A-D**) Same as on Figure 7. Initial nRT participation probability and TC spikes/burst show significant correlation with spindle length. Initial and final nRT participation probability also correlates significantly.
